# Design of Flexible Films Based on Kinked Carbon Nanofibers for High Rate and Stable Potassium-Ion Storage

**DOI:** 10.1007/s40820-022-00791-y

**Published:** 2022-01-22

**Authors:** Qiaotian Xiong, Hongcheng He, Ming Zhang

**Affiliations:** 1 Key Laboratory for Micro/Nano Optoelectronic Devices of Ministry of Education & Hunan Provincial Key Laboratory of Low-Dimensional Structural Physics & Devices, School of Physics and Electronics, College of Semiconductors (College of Integrated Circuits), Hunan University, Changsha, 410082 People’s Republic of China; 2 Semiconductor Technology and Application Innovation Institute of Changsha, Changsha, 410012 People’s Republic of China

**Keywords:** Flexible design, Finite element simulation, Sulfur-/nitrogen-Co-doped, Anode, Potassium-ion storage

## Abstract

With the emergence of wearable electronics, flexible energy storage materials have been extensively studied in recent years. However, most studies focus on improving the electrochemical properties, ignoring the flexible mechanism and structure design for flexible electrode materials with high rate capacities and long-time stability. In this study, porous, kinked, and entangled network structures are designed for highly flexible fiber films. Based on theoretical analysis and finite element simulation, the bending degree of the porous structure (30% porosity) increased by 192% at the micro-level. An appropriate increase in kinking degree at the meso-level and contact points in entanglement network at the macro-level are beneficial for the flexibility of fiber films. Therefore, a porous and entangled network of sulfur-/nitrogen-co-doped kinked carbon nanofibers (S/N-KCNFs) is synthesized. The nanofiber films synthesized from melamine as nitrogen sources and segmented vulcanization exhibited a porous, kinked, and entangled network structure, and the stretching degree increased several times. The flexible S/N-KCNFs anode delivered a higher rate performance of 270 mAh g^−1^ at a current density of 2000 mA g^−1^ and a higher capacity retention rate of 93.3% after 2000 cycles. Moreover, the foldable pouch cell assembled by potassium-ion hybrid supercapacitor operated safely at large-angle bending and showed long-time stability of 88% capacity retention after 4000 cycles. This study provides a new idea and strategy for the flexible structure design of high-performance potassium-ion storage materials.

**Supplementary Information:**

The online version contains supplementary material available at 10.1007/s40820-022-00791-y.

## Highlights

- Flexible films of kink porous carbon nanofibers are designed at the micro, meso and macro levels
- The fiber-film anodes with porous, kinked, and entangled network structures exhibit high rate and stability for potassium-ion storage

**Supplementary Information:**

The online version contains supplementary material available at 10.1007/s40820-022-00791-y.

## Graphical abstract

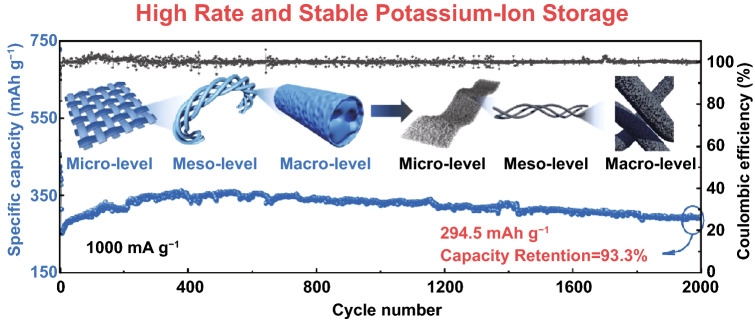

## Introduction

With the recent advancement in energy storage research on large-scale rigid equipment (energy vehicles), life quality has been significantly improved. But these developments have put forward higher requirements for monitoring human health indicators. Therefore, research on energy storage devices (ESDs) for portable and efficient flexible electronics (smart bracelets and virtual reality glasses) has attracted much attention [1]. As the main component of commercial ESDs, progress in lithium-ion batteries (LIBs) has been limited due to the crustal abundance of lithium. Potassium-ion batteries (PIBs) are considered as potential substitutes because of their high abundance, lower cost, closer to the standard potential of LIBs, and safer potassium potential (0.2 V) [2, 3]. To improve the flexibility and safety of ESDs for wearable electronics, a lot of research has been done on device structure design [4–6]: Woo et al. [7] used the electric displacement reaction to prepare the fabric electrode for integrated LIBs. Zhu et al. [8] used photographic printing technology to grow active materials on rice paper substrate as flexible electrodes. However, materials with flexible substrates have some limitations as well, such as low energy density, complex manufacturing process, high cost, and easy falling off of active materials. Therefore, pure carbon materials such as graphene, carbon nanotubes (CNTs), cellulose, and carbon nanofibers (CNFs) with good mechanical, physical, and chemical properties are suitable for self-supporting electrodes [4, 9–11, 12]. Zhang et al. [13] used graphene oxide with carbon quantum dots as a flexible anode plate for PIBs and achieved a specific capacity of 310 mAh g^−1^ at 100 mA g^−1^. Zen et al. [14] introduced CNTs in graphite foam carbon frames as free-standing anodes for PIBs, exhibiting a reversible capacity of 226 mAh g^−1^ after 800 cycles at 100 mA g^−1^. However, their energy density and cycle stability are still unsatisfactory for wearable devices [14].

The introduction of heteroatoms such as F, N, O, S, and P is a simple and efficient way to improve the electrochemical performance of carbon materials. Xu et al. [15] prepared nitrogen-doped CNFs for PIBs with excellent rate capability and cycling performance. Hu et al. [16] also confirmed that doping nitrogen and sulfur atoms can expand the graphite layer spacing and increase the structural defects and active sites, suitable for the efficient storage of potassium ions. However, the effects of heteroatoms and the doping process on the flexibility of single carbon nanofiber and films are rarely reported. In previously reported doped carbon nanofiber films, only inherent mechanical properties are investigated, without focusing on special design and morphology to improve the flexibility.

Herein, based on theoretical analysis and finite elemental simulation, basic sources and factors influencing the flexibility are analyzed and explained from the macro-, meso-, and micro-levels. The structural requirements include a porous structure with special pore distribution (high microporosity and appropriate internal mesoporosity), a kinked structure, and an entangled network with more contact points. Considering the mechanical and electrochemical requirements of flexible energy storage materials, sulfur-/nitrogen-co-doped kinked carbon nanofibers were prepared by electrospinning, annealing, and segmented vulcanization. After quantitatively adjusting the temperature, dose ratio, and optimizing the vulcanization process, triple special structures of S/N-KCNFs were formed at 700 ℃ with MA: PAN = 1:1. A large number of micropores and a few mesopores in the fiber, an obvious kinked structure, and an entangled network closer to the textile were observed. S/N-KCNFs exhibited excellent flexibility compared with other fibers without multiple flexible structures. By controlling the flexibility of the electrode, the electrochemical performance was also optimized. When S/N-KCNFs were used as anode material in PIBs, they showed high reversible capacity, high rate capacity, and excellent cycle stability (93.3% capacity retention after 2000 times). The characterization results show that N/S doping resulted in a larger graphite layer spacing, more defects, and activity centers, conducive to the intercalation reaction of potassium ions and the adsorption of pseudocapacitance effect. More importantly, the pouch cell PHSC based on S/N-KCNFs and treated activated carbon (TAC) demonstrated good flexibility and worked safely when folded. The designed multi-level analysis and design strategy for flexible nanofiber films can be applied in energy storage materials.

## Experimental Section

### 1Preparation of N-KCNFs, S-KCNFs, and S/N-KCNFs

Firstly, 0.72 g of PAN and 0.72 g of melamine (MA) were added to 6 mL N, N-dimethylformamide (DMF) and stirred in a water bath at 60 ℃ for 72 h to obtain a uniformly dispersed electrospinning solution. The precursor solution was then poured into a 10-mL syringe with 21# needles. The distance and voltage between positive and negative aluminum plates are 15 cm and 18 kV, and injection flow rate was fixed at 0.5 mL h^−1^. The pure white fiber films were heated for 2 h in air at a heating rate of 1 ℃ min^−1^ to 230 ℃. In order to obtain flexible N-doped carbon nanofiber mats with kinks (N-KCNFs), the brown films were heated at 700 ℃ for 2 h at a heating rate of 3 ℃ min^−1^. The pre-annealed product and sublimated sulfur were mixed in a combustion boat at a mass ratio of 1:3 and then heated to 155 ℃ at a heating rate of 1 ℃ min^−1^ in Ar atmosphere for two hours. Then, we keep it at 270 ℃ for 2 h to further decompose melamine and react with the remaining hydrogen sulfide gas, in order to make N-KCNFs fully react in hydrogen sulfide gas and then further heat to 700 ℃ for 2 h, in order to remove excess sulfur powder and make the target product S/N-KCNFs more stable. As a comparison, the melamine content of S/N-KCNFs-0.5 and S/N-KCNFs-2 samples is 0.5 and 2 times of the weight of PAN, respectively. Sulfur-doped kinked carbon nanofibers (S-KCNFs) were obtained by segmented vulcanization of nanofiber films without melamine.

### 2.2Electrochemical Measurement

A 12-mm-diameter adhesive-free self-supporting electrode was used for electrochemical testing. The mass range of the self-supporting electrode is 0.8–1.2 mg cm^−2^. Potassium foil with a diameter of 12 cm was used as counter electrode. A 3-M KFSI (potassium bis(fluorosulfonyl)imid) electrolyte was used to dissolve the electrolyte in dimethyl ether (DME) solvent. CR2032 coin-shaped battery shell was used to assemble a half-cell in a glove box filled with Ar. The constant current charge discharge cycle was measured with NEWARE battery tester in the potential range of 0.01–3.0 V (vs. K/K^+^) at constant current density of 0.05–2 A g^−1^. Cyclic voltammetry curve (CV, scanning rate range: 0.1–1.5 mV s^−1^) and electrochemical impedance spectrum (EIS, frequency range: 10 kHz–100 kHz) were recorded by CHI660E electrochemical workstation.

### Treatment of Activated Carbon

Activated carbon with high porosity was prepared by treating commercially activated carbon particles. Firstly, large particles of commercially activated carbon were ground into small particles, 500 mg of which were put in dilute nitric acid (about 5 mol L^−1^) solution and kept in 90 ℃ ventilated ovens for 5 h. The activated carbon was washed several times with deionized water to remove the residual nitric acid and other impurities. The precipitate obtained by centrifugation was dried in a vacuum drying oven at 60 ℃ for 12 h. Then, the activated carbon treated with nitric acid was heated at 900 ℃ for 2 h at the rate of 5 ℃ min^−1^ in Ar atmosphere, and finally, the high-porosity TAC was obtained.

### Preparation of Flexible Packaging Hybrid Devices

The treated activated carbon was further ground into fine powder and fully mixed with binder CMC and conductive agent carbon black in the ratio of 8:1:1. Then, it was evenly coated on the carbon-coated aluminum plate as the anode of the whole battery, and the quality control of TAC load was 2–4 times of that of cathode material. Before assembling the whole cell, it is necessary to pre-potassium the anode material: S/N-KCNFs anode is charged and discharged in half-cell at a constant current of 50 mA g^−1^ for five cycles (discharged to 0.01 V) and then washed in electrolyte solvent to remove the residual electrolyte salt. TAC smear electrode was used as the cathode of the whole battery, glass fiber was used as the diaphragm, and S/N-KCNFs was used as the self-supporting anode. The potassium-ion hybrid supercapacitor is encapsulated (PHSC) in aluminum plastic to obtain pouch cell. In this study, the voltage range of the PHSC is 0.1–4.0 V. The mass of active material used in current density calculation is the total mass of anode and anode material.

### Simulation and Calculation Process

In order to simplify the calculation and highlight the core research points, we simplified the fiber into an isotropic cylinder (radius(*R*) of 20 nm and length (*L*) of 666 nm). The material parameters of the solid in the cylinder are Poisson's ratio of 0.35, elastic modulus of 50 GPa, density of 1.75 g cm^−3^, and yield strength of 7.8 GPa [17, 18]. The micropores are reflected in different porosity percentages, and the internal mesopores are simplified into hollow spheres (radius(*r*) of 5.5 nm). These spheres are randomly distributed in the cylinder. In order to simplify the calculation and close to the experimental data, the porosity was set to 0%, 15%, and 30%. In the three-dimensional rectangular coordinate system, the cylinder is placed vertically on a horizontal plane, and the origin coincides with the center of the circle. As for the choice of theoretical model, generally speaking, carbon nanofibers with large diameter and length show better conductivity and flexibility. On the other hand, in engineering practice, the ratio of span length to section height (*L*/*H*) is much greater than 5. Therefore, more accurate results can be obtained by quoting Eq. (S1), so we set *L/H* as large as possible to eliminate the error of calculation results. The lower end of the cylinder is fixed at the origin, and a loading force (*F*) is applied at the upper end of the cylinder, where the direction of F is the positive direction of the x-axis. Gradually increasing the size of F makes the upper end of the cylinder move slowly and the whole fiber bend. The projection of the center coordinate of the end point on the cylinder on the x-axis is named vertical displacement, for the maximum stress that will be generated in the fiber at this time.

## Results and Discussion

### Theoretical Analysis of Flexibility

From one-dimensional fiber to a three-dimensional winding system, many factors influence flexibility [19, 20]. The mechanical strength of a single fiber determines the mechanical strength of the film [21]. The appearance of a single fiber is closer to the appearance of spring at a high-kink degree of fiber, whereas the contact point between fibers increases the friction transmission and diffusion inside the fiber membrane. Finally, an entangled network formed by three-dimensional disordered fibers further improves the overall flexibility. Therefore, we analyze the source of flexibility from three aspects: the micro-level (i.e., porous structure), meso-level (i.e., kink structure), and macro-level (i.e., entanglement network).

**Fig. 1:**
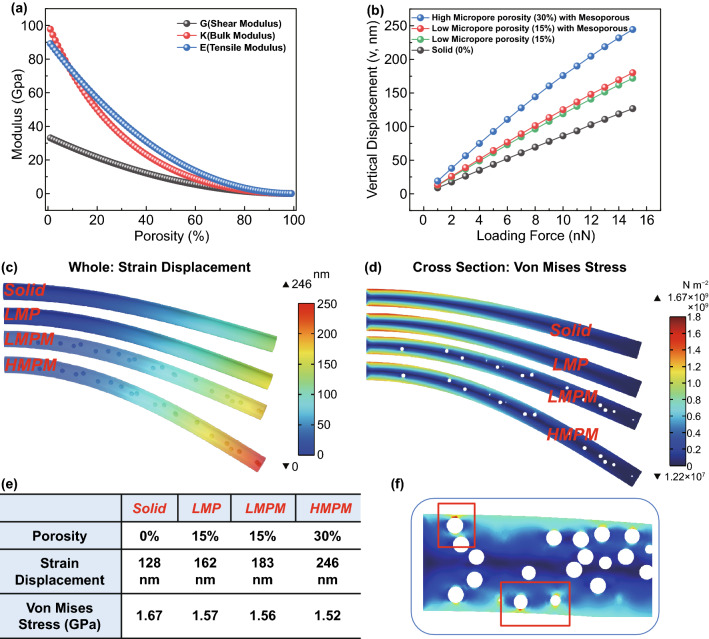
**a** The corresponding reference values of shear modulus, volume modulus, and elastic modulus under different porosities when the Poisson's ratio of raw materials is 0.35. **b** Vertical stress displacement of non-porous fiber (solid), low microporous fiber (LMP), low microporous fiber with mesoporous interior fiber (LMPM) and high microporous fiber with mesoporous interior fiber (HMPM) under different loading forces. When the loading force is 15 nN, the overall displacement diagram of solid, LMP, LMPM, and HMPM (**c)**, the sectional stress distribution (**d)**, and the specific parameter table (**e)** are presented. **f** Simulation model picture of stress singularity when excess mesoporous is located on the fiber surface

First of all, at micro-level, we focus on porous structure inside the fiber. Many theoretical calculations explain the change in elastic constant with porosity. Generally, when determining the porosity (*P*), the bulk modulus, shear modulus, and elastic modulus of the material are calculated by Eqs. (1–3) [22]:

1 $$\frac{G}{K} = \frac{3}{4} + \frac{{3\left( {1 - 5\mu _{m} } \right)}}{{4\left( {1 + \mu _{m} } \right)}}\left( {\frac{G}{{G_{m} }}} \right)^{{\frac{3}{5}}}$$

2 $$\frac{G}{{G_{m} }} = \left( {1 - c} \right)^{2}$$

3 $${\text{E}} = \frac{{9{\text{KG}}}}{3K + G}$$

where *G*_m_ is the shear modulus and ${\mu }_{m}$ is the Poisson's ratio of original material. The porosity alters the flexibility of material by changing the modulus values of materials. In energy storage materials, it is theoretically feasible to improve the flexibility of carbon nanofibers by changing their porosity (Figs. 1a and S1). We further simulated and calculated the isotropic carbon nanofibers with different flexibilities by changing the porosity via finite element analysis. In the simulation experiment, after simplifying the fiber into a slender cylinder, we used a simplified cantilever beam model for analysis. Non-porous fiber (0% porosity) (solid), low microporous (15% porosity) fiber (LMP), low microporous (15% porosity) fiber with mesoporous interior (LMPM), and high microporous (30% porosity) fiber with mesoporous interior (HMPM) are set, respectively. The setting of HMPM is due to the presence of mesopores, facilitating the electrode expansion. Therefore, a larger mesoporous model is introduced into this calculation. In addition, stress singularity during bending is the reason for the design of mesopores inside the fiber rather than on the fiber surface, resulting in uncontrolled stress and crack in the fibers (Figs. 1f and S2). With a slow increase in external load force, the unfixed end of the fiber moves in the direction of load force. Figure 1b shows that the fiber end displacement increases with the increase in porosity when the given load force is consistent, indicating better flexibility. The HMPM with a small number of mesopores inside has the best flexibility. Figures 1c and S3 are the schematic diagrams of the strain displacement of each simulated nanofiber at a 15-nN loading force. The bendability of the HMPM fiber model increases by 192% compared to that of the solid fiber model. To ensure the tensile strength in the bending process, the strain of the fiber is considered. The place where the maximum strain stress occurs indicates the fracture and failure points. In addition, a larger value of maximum strain stress indicates that fracture is easier. None of the four materials reached the bending strength of the material, as shown in Figs. 1d–e and S4. These results indicate that an increase in porosity reduces the shear modulus and volume modulus of the material, resulting in better flexibility.

Inspired by the spring, we focus on the kinked structure at the meso-level. The diameter of a single fiber and crimp factor directly influences the flexibility of the fiber films [23, [24]. The flexibility ratio (*F*_R_) of straight and curved fiber of the same length reflects the improved flexibility of kinked fiber (Eq. (4)).

4 $$F_{R} = 2k\beta = \frac{2E \times A \times \beta }{{d_{0} }}$$

where *k* (N m^−1^) is the fiber axial stiffness of the spring, and *β* is the radius of curvature (R), and d_0_ is the distance between adjacent connection points. In fiber with the same modulus (*E*) and cross-sectional area (*A*), a higher degree of kink is observed in the fibers with a higher radius of curvature and a shorter distance between the connection points (Fig. S5), indicating the higher flexibility of fibers. In energy storage materials, twisting the slender carbon nanofibers into a complete spring structure is very difficult. Therefore, the flexibility of fibers is increased by appropriately increasing the number of kinks or shortening the kink spacing, without affecting other physical properties such as conductivity and electrolyte wettability.

At the macro-level, the disordered interweaving of fibers forms an entanglement network. It is a difficult and key factor in investigating the friction work between contact points in fibers with an entangled network [25], as shown in Eqs. (S6–S8) [26]. When the number of contact points in the entanglement network is large (i.e., close to the fabric state), the mechanical properties in the material have obvious hysteresis, indicating that textile analogs can buffer the deformation of fiber films under external load. However, in energy storage materials, the direct use of textile products is not suitable because increasing the contact point in the entanglement network ensures sufficient electrochemical activity. Flexibility electrode materials alleviate their deformation. In addition, when the flexible energy storage device is deformed, the electrode also protects the adjacent important elements, such as the separator. The requirements for improving the flexibility at the meso-level are similar to those at this level [24]:

5 $$E_{{{\text{cf}}}} = \frac{{2\rho_{f} E_{{{\text{mat}}}} t_{{{\text{mat}}}} }}{{m/(W_{{{\text{mat}}}} L_{{{\text{mat}}}} )}}$$

where the basic width (${w}_{\mathrm{mat}}$), length (${l}_{\mathrm{mat}}$), and thickness ${(t}_{\mathrm{mat}})$, Young's modulus (${E}_{\mathrm{mat}}$), and fiber density (${\rho }_{f}$) of the fiber films are known; the ${E}_{\mathrm{cf}}$ of bent fiber is expressed by Eq. (5). At fixed diameter, the larger curl factor indicates knitting of non-woven fabric with better flexibility.

From one-dimensional fiber to a three-dimensional winding system, many factors influence flexibility [19, 20]. The mechanical strength of a single fiber determines the mechanical strength of the film [21]. The appearance of a single fiber is closer to the appearance of spring at a high kink degree of fiber, whereas the contact point between fibers increases the friction transmission and diffusion inside the fiber membrane. Finally, an entangled network formed by three-dimensional disordered fibers further improves the overall flexibility. Therefore, we analyze the source of flexibility from three aspects: the micro-level (i.e., porous structure), meso-level (i.e., kink structure), and macro-level (i.e., entanglement network).

### Synthesis and Characterization of Materials

**Fig. 2:**
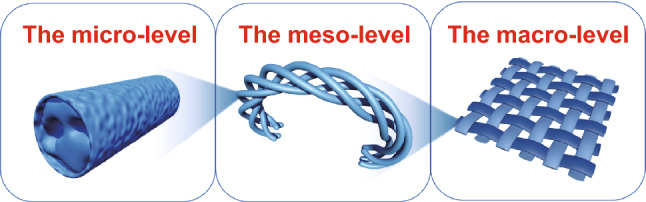
The figure shows the source of flexibility from three aspects: The micro-level (i.e., porous structure), meso-level (i.e., kink structure), and macro-level (i.e., entanglement network)

**Fig. 3:**
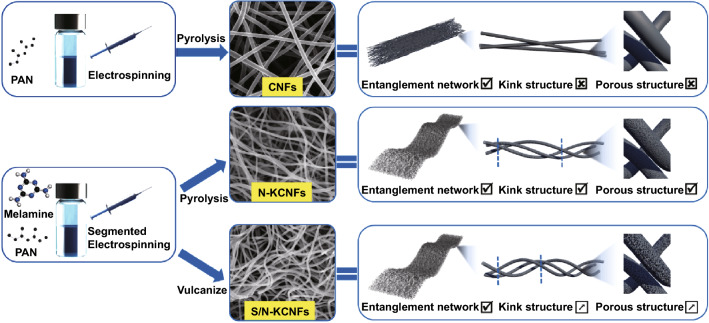
Schematic diagram of preparation of CNFs, N-KCNFs, and S/N-KCNFs. And judging from three levels of influencing factors of flexibility, in which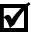 'tick' means available, 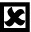
'cross' means not available, and **↗** means increased

**Fig. 4:**
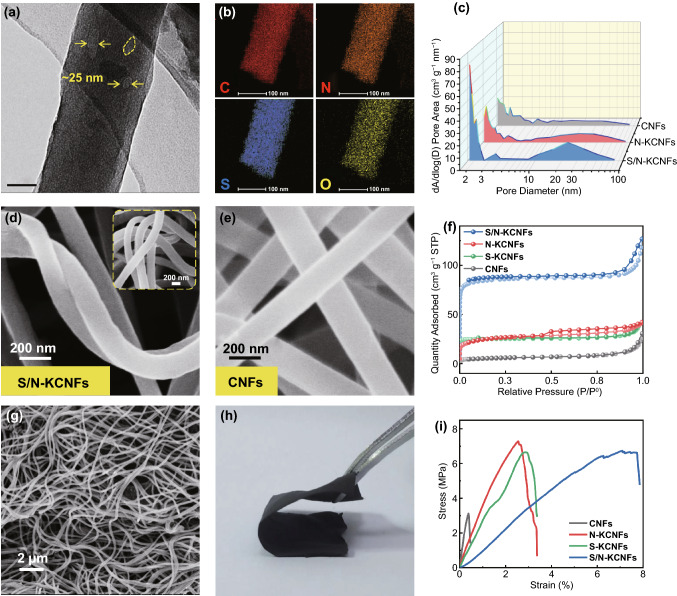
**a** TEM image, **b** EDS element mapping (C, N, O, and S elements), and **d, g** SEM image corresponding to S/N-KCNFs; **e** SEM image of CNFs; **c** the pore size distribution and **f** nitrogen adsorption–desorption isotherms curve of S/N-KCNFs, N-KCNFs, S-KCNFs and CNFs; **h** digital photographs of S/N-KCNFs bending; **i** The stress–strain curves of S/N-KCNFs, N-KCNFs, S-KCNFs, and CNF films

The porous properties of carbon fibers improve the flexibility at the micro-level, whereas the kink structure at the meso-level results in a high flexibility. At the macro-level, the carbon fiber films with entanglement networks show excellent flexibility (Figs. 2 and S6). According to these results, a sulfur- and nitrogen-doping strategy is proposed to achieve the special structure for high-flexible carbon fiber films (Fig. 3). Firstly, melamine (MA) and polyacrylonitrile (PAN) are fully mixed and then annealed to obtain N-KCNFs, followed by segmented vulcanization to form S/N-KCNFs. At the micro-level, fibers with a diameter of about 100–120 nm are prepared (Fig. 4a), which are characterized by transmission electron microscopy (TEM). The energy-dispersive spectrometer (EDS) shows that nitrogen and sulfur are successfully incorporated in the carbon nanofibers (Fig. 4b) and are uniformly distributed. The adsorption characteristics of fiber films are studied by nitrogen adsorption–desorption isotherm. The porous structure of S/N-KCNFs, N-KCNFs, and CNFs is obtained by BJH desorption $\mathrm{dA}/\mathrm{dlog}(\mathrm{D})$ Pore Area as shown in Fig. 4c. Compared with the other two materials, S/N-KCNFs have more micropores (less than 2.5 nm) and mesoporous (about 25 nm in diameter). TEM images (Figs. 4a and S5) also confirmed the special pore distribution structure, a large number of micropores with a few mesopores inside. This is mainly due to the change in the internal morphology of fibers after adding melamine [27]. The special distribution of micropores and mesopores is suitable for the wettability of electrolytes and the electrode volume expansion caused by potassium-ion insertion/extraction during the charge and discharge process [27]. The S/N-KCNFs show a typical microporous-type IV curve (Fig. 4f), with a very high BET surface area of 340 m^2^ g^−1^, much higher than that of S-KCNFs (92.5 m^2^ g^−1^), N-KCNFs (83 m^2^ g^−1^) and KCNFs (13 m^2^ g^−1^). The decomposition of melamine releases CO_2_, NH_3_, and other gases, thereby forming a microporous structure [27]. The CNFs with the smallest surface area and the kink-free structure correspond to the “solid’ in the model. The N-KCNFs and S-KCNFs with increased specific surface area and no mesoporous inside correspond to the LMP in the model. The S/N-KCNFs have micropores and mesopores with a diameter of about 25 nm, corresponding to the LMPM in the model (Fig. 1c–e). The segmented vulcanization is also a reason for high specific surface area [28] as it incorporates flexibility in the fiber microstructure, similar to the porous sponge structure where soft sponge not only has microporous structure but also has large mesopores inside [21]. At the meso-level, the self-bending kink structure (similar to small spring) is observed by scanning electron microscope (Fig. 4d). Compared with N-KCNFs, S-KCNFs, and CNFs (Fig. S8), S/N-KCNFs have a larger rotation factor (Eq. (4). At the macro-level, SEM images (Fig. 4g) show entanglement networks with large aspect ratios and disorderly interwoven morphology, similar to textile fabrics. The formation in this structure is explained as follows: Firstly, because nitrogen-rich melamine is uniformly dispersed in the electrospinning solution, a large amount of C_3_N_4_ is formed in and around the fiber during annealing [29]. Secondly, melamine is not soluble in DMF solvent, so segmented electrospinning is used for better mixing of melamine with PAN carrying out a special chemical reaction [27, 30]. Thirdly, the step heating method is for annealing to activate the cyano-groups in PAN chain and react with the active amino group in melamine during carbonization and vulcanization, to form a stable kink structure [31]. The bending test in Fig. S9 reveals that the S/N-KCNFs films’ electrode sheet can withstand 180º bending and folding, and quickly returns to its original state after kneading. More importantly, the bending test shows that the fiber exhibits excellent flexibility (Fig. 4h). Furthermore, the stress–strain test shows that the S/N-KCNFs have more than 8 times tensile deformation compared with ordinary carbon nanofibers (Fig. 4i) [32]. This shows that the test results are consistent with the theoretical simulation results. Among them, the internal special porous distribution structure of S/N-KCNFs contains both micropores and some mesopores, which conforms to the setting of LMPM model with micropores and internal mesopores in theoretical simulation and is considered to have appropriate flexibility and mechanical properties. This strategy of designing the flexible fiber films at the micro-, meso- and macro-levels is feasible and effective, and S/N-KCNFs with the triple special structure are successfully applied in energy storage materials to meet the requirements of flexible design.

In this study, the morphology of fibers (including porosity, kink, and the contact points of entanglement networks) is controlled and optimized by quantitatively adjusting the doping of nitrogen and sulfur. SEM images of S/N-KCNFs-0.5, S/N-KCNFs, and S/N-KCNFs-2 are obtained. At different mass ratios of MA: PAN, the morphology of the micro helix structure differs significantly (Fig. S10). With increasing proportion, more phase separation and caked carbon impurities around the electrospun fibers occur, but too much phase separation is not conducive to the friction transfer and rapid electron conduction, resulting in poor electrochemical performance. The N content is analyzed by X-ray photoelectron spectroscopy (XPS), as shown in Table S1. The XPS results show that the nitrogen content in the target sample increases with the increase in the mass ratio of MA in the pre-spinning solution. The flexibility of samples with different mass ratios also differs significantly. The presence of excessive non-fibrous (scattered particles without fixed appearance) carbon hinders the diffusion of friction between fibers and reduces the flexibility, resulting in cracking of the fiber membrane. Table S2 shows that changing the annealing temperature (600/700/800 ℃) also has an important effect on nitrogen content, consistent with previous reports [33]. The heteroatom content and graphitization degree play a vital role in determining the number of active sites, affecting the storage performance of potassium ions. Therefore, we ensured its electrochemical performance while considering the flexibility design, so the carbonization temperature is set at 700 ℃, and the mass ratio of MA: PAN is selected as 1:1.

**Fig. 5:**
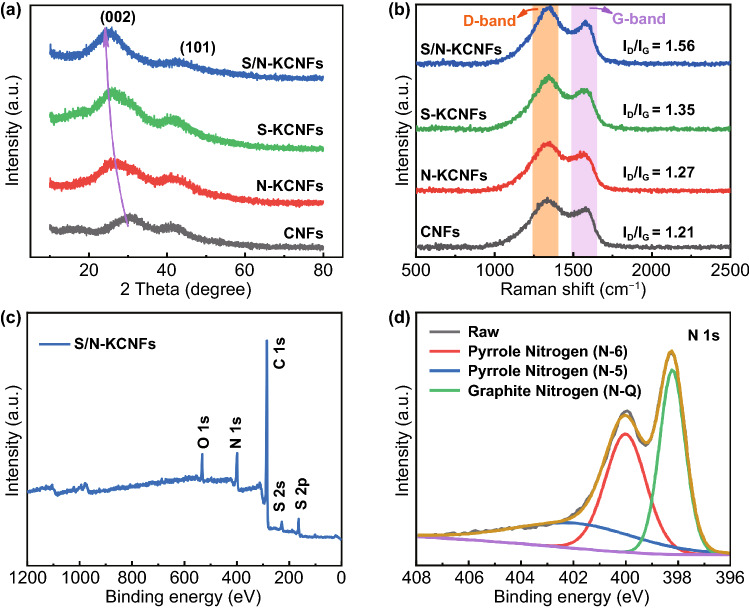
**a** XRD spectra and **b** Raman spectra of S/N-KCNFs, S-KCNFs, N-KCNFs and CNFs; **c** XPS measurement spectra of S/N-KCNFs; **d** high-resolution N 1 s spectra of S/N-KCNFs

To further study the internal structure, the crystal phase characteristics of S/N-KCNFs, S-KCNFs, N-KCNFs, and CNFs are studied by X-ray diffraction (XRD) as shown in Fig. 5a. All four samples exhibit two distinct broad diffraction peaks at 26º and 43º, corresponding to the (002) and (101) crystal planes of classical graphite carbon, respectively. The wider diffraction peaks indicate a more disordered carbon structure. The (002) peaks of S/N-KCNFs, S-KCNFs, N-KCNFs, and CNFs are 23.32º, 24.33º, 24.53º, and 26.79º, respectively. According to Bragg's law, the interlayer spacing of the (002) plane is 3.8, 3.69, 3.65, and 3.39 Å, respectively. Nitrogen sulfur co-doping shows more obvious shift than single-atom doping, which can be explained by the synergistic effect between nitrogen and sulfur dopants. In fact, the heteroatom N replaces the carbon atom of the C–C bond and maintains the non-bonded state. Therefore, the nitrogen atom adopts the pyridine environment, while the sulfur atom bonds with two carbon atoms and adopts the edge bonded state [34]. The increase in layer spacing is attributed to the introduction of nitrogen or sulfur heteroatoms with a larger radius than carbon atoms in the carbon lattice, causing a large number of carbon defects. These results are consistent with TEM and EDS results. The R values of S/N-KCNFs, S-KCNFs, N-KCNFs, and CNFs are 1.75, 1.96, 2.02, and 2.26, respectively, indicating the contribution of heteroatoms to carbon structural defects (Fig. S11). Raman spectroscopy is further used to investigate the disorder degree of carbon structure in all four samples (Fig. 5b). Two characteristic broad peaks are located at 1345 and 1579 cm^−1^, usually interpreted as the D-band interpreted as hexagonal carbon ring breathing mode and the G-band caused by C = C chemical bond stretching of C-*sp*^2^, also considered a characteristic peak of graphite arrangement [35]. To quantify the defects in carbon materials, D-band-to-G-band intensity ratio (*I*_D_/*I*_G_) is also considered an important parameter [36]. It is worth noting that the peak intensity ratio of S/N-KCNFs (1.56) is higher than that of S -KCNFs (1.35), N-KCNFs (1.27), and CNFs (1.21) indicating the structural defects caused by the doping of nitrogen and sulfur atoms. X-ray photoelectron spectroscopy (XPS) is used to study the surface chemical properties of S/N-KCNFs, S-KCNFs, and N-KCNFs (Figs. 5c and S12–S13). Table S3 shows the atomic percentage of elements in S/N-KCNFs, S-KCNFs, and N-KCNFs. Results indicate that nitrogen and sulfur atoms are successfully doped in the carbon structure, with a relatively high nitrogen doping ratio of 13.3%. In the C 1 s spectrum, four characteristic peaks correspond to C–C (284.72 eV), C–N (285.71 eV), C = O (286.95 eV), C–O/C–S (288.74 eV), indicating that the material is still dominated by carbon, and nitrogen and sulfur atoms are only used as dopants. The N 1 s spectra of S/N-KCNFs show three characteristic peaks of pyrrole nitrogen (N-5), pyrrole nitrogen (N-6), and graphite nitrogen (N–Q) corresponding to 398.2, 399.9, and 401.2 eV, respectively (Fig. 5d) [37]. The edge nitrogen accounts for 70% of the total nitrogen content. According to a previous report, edge nitrogen is beneficial to the electrochemical activity of the material, while graphitic nitrogen has a weakening effect on conductivity [38]. In high-resolution XPS, O 1 s spectra are fitted as C–O (530.20 eV), C = O (532.31 eV), and O-S (528.28 eV), and S 2p spectra are fitted as S 2p_2/3_ (163.80 eV), S 2p1/3(164.98 eV), and sulfate (167.28 eV) [39]. High heteroatom doping rate leads to a high defect degree in carbon materials causing expansion of graphite interlayer distance (also verified by XRD and Raman Spectra) as shown in Fig. S13f. The addition of nitrogen and sulfur not only introduces special pore distribution and high specific surface area but also optimizes the internal structure of carbon nanofibers by increasing the active center and defect degree and expanding the layer spacing [40]. This feature leads to the rapid insertion/extraction of potassium ions, facilitating higher reversible capacity as flexible anode materials.

### Potassium Storage Performance

**Fig. 6:**
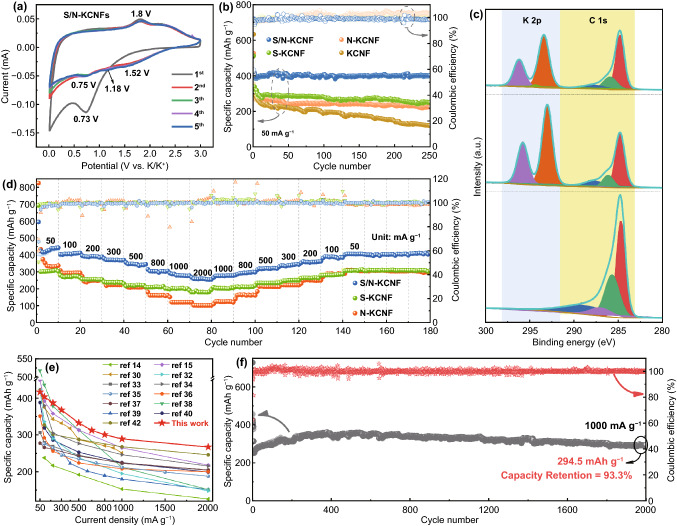
**a** The first five CV cycles of S/N-KCNFs; **b** the specific capacity cycling test and coulomb efficiency were carried out at current density of 50 mA g^−1^, when S/N-KCNFs, S-KCNFs, N-KCNFs and CNFs are used as anodes of PIBs; **c** the ex situ XPS of S/N-KCNFs as anode of PIBs during discharge and charging; **d** the rate capability of S/N-KCNFs, S-KCNFs and N-KCNFs at 50–2000 mA g^−1^ current density; **e** comparison of the rate performance of different carbonaceous materials in PIBs with our current work; **f** the steady cycle performance and coulomb efficiency of S/N-KCNFs under high current density of 1000 mA g^−1^ for 2000 ultra-long cycles were investigated

To further investigate the electrochemical performance of S/N-KCNFs, a half-cell is assembled with potassium foil as reference electrode and negative electrode, S/N-KCNFs as an anode, and 3 M KFSI as electrolyte. Firstly, the electrochemical performance S/N-KCNFs carbon materials is studied by cyclic voltammetry. Figure 6a shows the first five cycles of the CV curve with a scan rate of 0.1 mV s^−1^ and a voltage range of 0.01–3 V. An obvious reduction is observed at 0.73 V in the first circle, which suggests the decomposition of electrolytes and the formation of a stable solid electrolyte interface (SEI film) on the electrode surface. This reduction, known as irreversible reduction peak, disappears in the subsequent cycle, indicating the promising stability of SEI film after the first cycle, without decomposition, cracking, and regeneration. The sharp reduction at 0.01 V is associated with the formation of KC_8_ formed by potassium-ion intercalation between graphite layers. Similar characteristic peaks are also observed in N-KCNFs and S-KCNFs, indicating the formation of SEI film and the formation of potassium graphite intercalation compounds such as KC_8_. In subsequent cycles, three reduction peaks are observed at 1.52, 1.18, and 0.75 V associated with S–C_x_–N- formation [35], the chemical reaction between potassium ions and N-5, N-6, and the reaction between potassium-ions and unsaturated sulfur atoms active center, respectively. During the cathode scanning of N-KCNFs (Fig. S19), the wide peak of about 0.43 V in the subsequent discharge should be related to the reversible potassium process in the active site introduced by nitrogen [34, 41]. During the charging process, an oxidation peak at 1.8 V is closely related to the reaction between potassium ions and doped S atoms and the deintercalation of potassium ions [35]. The following CV curves gradually tend to overlap and completely overlap at the beginning of the third cycle, indicating that the electrode material has good cyclic reversibility. These reactions are also justified ex situ XPS (Figs. 6c and S14). In addition, through the constant current charge–discharge cycle test (Figs. 6b and S15), the specific capacity of S/N-KCNFs half-cell remains at 410 mAh g^−1^ after 100 cycles, much higher than that of N-KCNFs electrode half-cell (300 mAh g^−1^). The first charge–discharge coulomb efficiency (ICE) is 66.1%, and it remains at 96.2% after 250 cycles. It also shows that the material has excellent cycle reversibility. Figures 6d and S16 show the rate performance of the S/N-KCNFs electrode half-cell and N-KCNFs electrode half-cell. The specific charging capacities are 410, 380, 350, 340, 320, 300, and 270 mAh g^−1^ at a current density of 50, 100, 300, 400, 500, 800, 1000, and 2000 mA g^−1^. When the current recovers to 50 mA g^−1^, the charge–discharge capacity also recovers to the original 410 mAh g^−1^. These results show that the material demonstrates excellent rate performance as anode material for PIBs. Compared with the reported carbon-based PIBs anode materials, the S/N-KCNFs demonstrated relatively higher rate performance (Fig. 5e) [17, 37, 40, 44–52]. In addition, the specific capacity is 330 mAh g^−1^ at a constant current of 1000 mA g^−1^ and the capacity retention rate reaches 93.3% after 2000 cycles of charge–discharge cycle test (Figs. 6f and S17). This excellent cycle stability is attributed to the special pore distribution of mesopores and micropores in the fiber, which is conducive to the full wetting of electrolyte and the volume expansion of electrode materials [27].

**Fig. 7:**
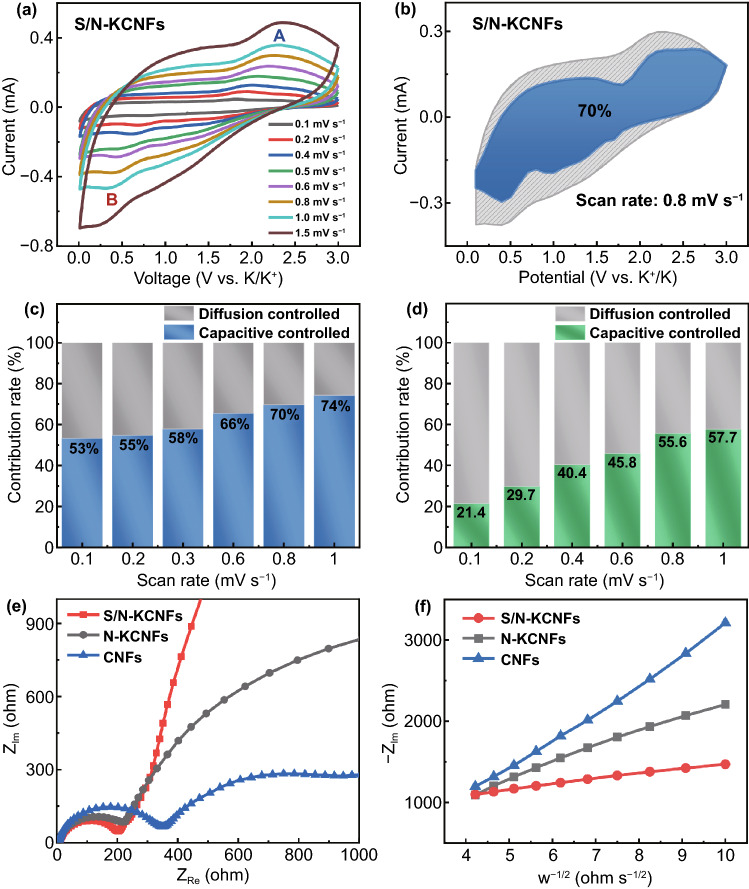
**a** CV curve of S/N-KCNFs at different scanning rates in the range of 0.1–1.5 mV s^−1^; **b** the CV curves of S/N-KCNFs show pseudocapacitance and diffusion-contribution at 0.8 mV s^−1^; the relative contributions of capacitance and diffusion-controlled charge storage of **c** S/N-KCNFs and **d** N-KCNFs at different scan rates are analyzed; **e** electrochemical impedance spectroscopy (EIS) (Nyquist plots), and **f** the slope corresponding to the linear relationship between ${\mathrm{Z}}_{Im}$ and ω of S/N-KCNFs, N-KCNFs and CNFs before cycling

To investigate the storage mechanism and electrochemical reaction kinetics of potassium ions, we carried out cyclic voltammetry analysis at different scan rates (0.1–1.5 mV s^−1^) and calculated the contribution of surface capacitance. As shown in Fig. 7a, with increasing scan rate, the CV curves of S/N-KCNFs are very similar, but the anodic peak has a gradual left shift and the cathode peak has a gradual right shift, indicating the polarization effect during the cycle. The pseudocapacitance effect is quantitatively analyzed according to the power-law relationship between peak current (I) and scanning rate (V) (Eq. (6)) [51]:

6 $$i = av^{b}$$

where *a* and *b* are constants. The value of *b* is obtained by calculating the slope according to the perfect fitting curve in Fig. S18a-b. When *b* is greater than 0.5 and close to 1, it indicates that the storage process is dominated by surface capacitance adsorption, while the *b* value equal to or less than 0.5 indicates that the storage process is dominated by Faraday embedding rather than surface capacitance adsorption. Compared with N-KCNFs ($b = 0.74$, $b = 0.74$), surface capacitance storage is the main storage in S/N-KCNFs ($b_{A} = 0.88,b_{B} = 0.97$), attributed to the high defect degree and high specific surface area. Therefore, the S/N-KCNFs electrode has faster electrochemical kinetics. To quantitatively separate the proportion of the pseudocapacitance effect and the diffusion Faraday embedding in the energy storage process, the pseudocapacitance contribution is calculated by Eqs. (7–8) [27]:

7 $$i\left( V \right) = k_{1} v + k_{2} v^{1/2}$$

8 $$i\left( V \right)/v^{1/2} = k_{1} v^{1/2} + k_{2}$$

where *k*_1_ and *k*_2_ are related constants. The first term on the right of Eq. (7) represents the pseudocapacitance control process, and the second term indicates the diffusion embedding control process. Figure S18c shows the slope k_1_ at different potentials, indicating high reliability of the slope obtained by high fitting degree. Figures 7b and S19 show the CV curves of S/N-KCNFs and N-KCNFs at 0.8 mV s^−1^, respectively. The boundary of the blue part in the figure is a typical pseudocapacitance CV curve. The integral of the area of the pseudocapacitance CV curve and the original CV curve are the capacities provided by the pseudocapacitance and the total capacitance, respectively. The contribution rate of pseudocapacitance is 70% at a 0.8 mV s^−1^ scan rate. Change in the pseudocapacitance contribution percentage is observed when the scan rate increases from 0.1 to 1.5 mV s^−1^ (Fig. 7c), which are 53%, 55%, 58%, 66%, 70%, and 74%, respectively. With increasing scan rate, the pseudocapacitance contribution percentage increases, indicating that the pseudocapacitance provides the main capacity in constant current charge–discharge and a high current density cycle. In addition, S/N-KCNFs have excellent rate performance as anode material of PIB. In contrast, the CV curve of the N-KCNFs electrode at different scan rates has a smaller area (Fig. 7d), indicating its capacity as an electrode. On the other hand, the contribution rate of pseudocapacitance is only 21.4% which increases to 57.7%, suggesting a slower charge–discharge chemical reaction compared with the S/N-KCNFs electrode. In addition, the respective capacitance contributions of S-KCNF and CNF electrodes are shown in Figs. S20 and S21. The electrochemical impedance spectrum (EIS) of half-cells with different flexible materials (Fig. 7e) shows that S/N-KCNFs exhibit minimum impedance. The smaller impedance is attributed to the addition of sulfur, more pyridine nitrogen, pyrrole nitrogen, and less graphite nitrogen, improving the electron transfer rate of PIBs [47]. The abscissa is ${\mathrm{Z}}_{\mathrm{Re}}$ and the ordinate is${\mathrm{Z}}_{\mathrm{Im}}$. The potassium-ion diffusion coefficient D_K_ of S/N-KCNFs is further studied by EIS in Eq. (9) [36, 52]:

9 $$D_{K} = \frac{{0.5R^{2} T^{3} }}{{A^{2} n^{4} F^{4} C^{2} \sigma^{2} }}$$

where *R* is the gas constant (8.314), T is the temperature (298 K), A represents the contact surface area between electrolyte and electrode (~ 0.8 cm g^−1^), n is the number of transferred electrons (*n* = 1), F is the Faraday constant (96,500 C mol^−1^), C represents the bulk density (~ 0.8 × 10^–3^), and σ is the Warburg coefficient, which corresponds to the slope obtained by fitting the linear relationship between ${\mathrm{Z}}_{Im}$ and ω of a half-cell (Fig. 7f). The diffusion coefficient of potassium ions in the electrolyte is calculated from the slope of $-{\mathrm{Z}}_{\mathrm{Im}} vs. {\omega }^{-1/2}$ or ${\mathrm{Z}}_{\mathrm{Re}} vs. {\omega }^{-1/2}$ curve [53]. The calculated diffusion coefficients for S/N-KCNFs (*σ* = 64.7), N-KCNFs (*σ* = 192.7), and KCNFs (*σ* = 345.6) are 3.18 × 10^–8^, 3.5 × 10^–9^, and 1.2 × 10^–9^, respectively. The diffusion of potassium ions in S/N-KCNFs is much faster, improving the rate performance of PIBs. The optimized pseudocapacitance effect, caused by a large number of active sites, defects, higher layer spacing, and large specific surface area is attributed to nitrogen and sulfur co-doping in carbon-based materials, facilitating the potassium ions adsorption its surface. It is worth noting that firstly, N/S co-doping brings higher K-adsorption ability, and the expansion of layer spacing can make potassium ions mobility/diffusion more rapidly in the carbon layer and then enhance the conductivity, so as to improve its storage capacity for potassium ions [41, 42, 46, 47]. The study of potassium storage kinetics shows that one is based on the chemical reaction of Faraday redox reaction with carbon to obtain KC_8_ for potassium storage, and the other is the pseudocapacitance effect to insertion/extraction to store potassium. The synergistic effect of the above two potassium storage kinetics leads to the excellent potassium storage performance of S/N-KCNFs, including high specific capacity, rate performance and ultra-long stability. Therefore, in addition to the analysis of the influence of nitrogen and sulfur co-doping on flexibility, nitrogen and sulfur co-doping plays an important role in improving potassium storage performance.

**Fig. 8:**
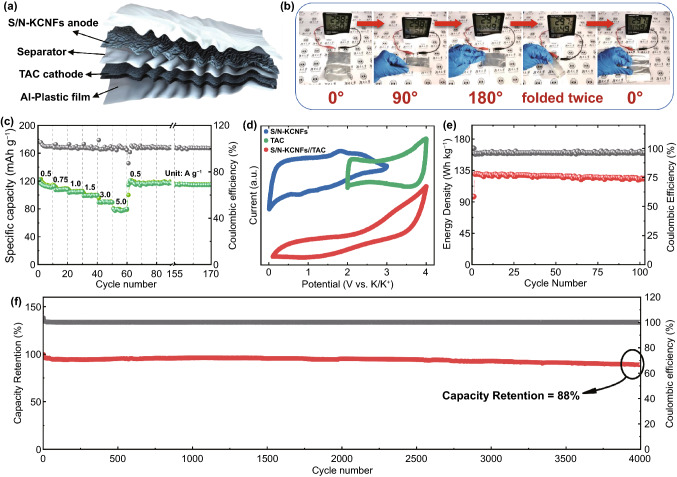
**a** Schematic illustration of hybrid capacitor S/N-KCNFs//TAC supplying power to wearable flexible devices; **b** digital photographs show that the pouch cell remains normal during the folding process (be bent from 0º to 90º, then folded to 180º, folded again, and then restored) to power the temperature and humidity electronic clock; **c** the rate capability of TAC at 0.5–5.0 A g^−1^ current density; **d** the CV curve of S/N-KCNFs, TAC, and S/N-KCNFs//TAC; **e** the energy density and Coulomb efficiency of S/N-KCNFs//TAC, respectively, with a power density of 270 W Kg^−1^ under 100 cycles; **f** the specific capacity and Coulomb efficiency of S/N-KCNFs//TAC under constant current charge and discharge of 10 A g^−1^ under 4000 cycles

The flexibility and electrochemical performance of S/N-KCNFs are verified by preparing a flexible pouch cell PHSC. The hybrid supercapacitor consists of two electrodes: the Faraday electrode and the capacitor electrode. In our experiments, S/N-KCNFs are used as the Faraday electrode and TAC as its capacitive adsorption electrode (the mass ratio of cathode/anode is 3:1), named S/N-KCNFs//TAC (Fig. S22) [54]. The schematic diagram of the foldable pouch cell is shown in Fig. 8a. The soft pouch cell is bent from 0º to 90º, then folded to 180º, folded again, and then restored. In this process, the flexible pouch cell works normally (provided power for the electronic clock) (Fig. 8b). Results show that S/N-KCNFs have the flexibility to be used as foldable electrodes. In addition, TAC is coated on the carbon-coated aluminum foil as the anode material. The CV curve, galvanostatic charge–discharge cycle performance, and morphology before and after cycling are studied by SEM. TAC is activated by dilute nitric acid and high temperatures. In addition, TAC has porous morphology and a strong adsorption capacity (Fig. S23). The rate capability diagram of the TAC button battery in the constant current charge–discharge cycle at 0.5, 0.75, 1.0, 1.5, 3.0, and 5.0 A g^−1^ shows high capacitances of 113, 108, 104, 99, 89.6, and 77 mAh g^−1^ (Fig. 8c). The CV curves of S/N-KCNFs electrode half-cell, TAC electrode half-cell (voltage window is 2.0–4.0 V), and the PHSC based on S/N-KCNFs//TAC (voltage window is 0.1–4.0 V) are shown in Fig. 8d. In addition, the battery with a constant current density charge–discharge cycle is tested and obtained the energy density is 124.9 Wh kg^−1^ after 100 cycles at 270 W kg^−1^ (Fig. 8e) and still maintained 78.5 Wh kg^−1^ even at 8360 W kg^−1^ (Fig. S24). At a maximum current density of 10 A g^−1^, the capacity retention rate is still as high as 88% after 4000 cycles (Fig. 8f). SEM images of disassembled battery (Fig. S25) show that the high-density SEI film formed on the surface of fiber films did not break or crack, another reason for the excellent cycling performance. Since the TAC electrode is prepared by a coating method, the soft anode material reduces the negative effects of material falling off during PHSCs bending. In general, the carbon nanofiber films with a special structure based on multi-level design strategy not only have high flexibility but also serve as anodes in practical flexible pouch cell of PHSCs.

## Conclusion

Sulfur-/nitrogen-co-doped kinked carbon nanofiber films were successfully prepared by electrospinning and segmented annealing strategy. The S/N-KCNFs exhibited porous, kinked, and entanglement network structures. At the micro-level, it showed high microporosity and a few mesopores, which increased the flexibility by changing the elastic modulus, the contact area with electrolyte, and volume expansion. At the meso- and macro-levels, the kink structure optimization improved the flexibility of a single fiber and increased the contact point of the entanglement network, forming a shape closer to textile materials. The simulation results showed that the flexibility of HMPM was 192%, higher than that of solid fiber at 15-nN load force. In the overall test, the S/N-KCNFs also showed excellent flexibility, which is 8 times that of ordinary carbon nanofibers. The introduction of nitrogen and sulfur dopants not only improved their flexibility but also enhanced their electrochemical activity. Compared with other doped carbon materials, the S/N-KCNFs showed high reversible capacity (407 mAh g^−1^) after 300 cycles at 50 mA g^−1^, high rate capacity (270 mAh g^−1^ at 2 A g^−1^), and long cycle stability (294 mAh g^−1^ high capacity after 2000 cycles at 1 A g^−1^). This can be explained by the increase in graphite layer spacing, defects, and active centers caused by nitrogen/sulfur dopants, conducive to the insertion/extraction of potassium-ion and pseudocapacitance effect. Furthermore, the pouch cell PHSC worked normally under different bending angles showing broad application prospects in flexible electronics. In general, this paper provides a new research perspective for the design and manufacture of flexible energy storage materials.

## Supplementary Information

Below is the link to the electronic supplementary material.

Supplementary file1 (PDF 2448 kb)
