# Supplementary material for: Design of Flexible Films Based on Kinked Carbon Nanofibers for High Rate and Stable Potassium-Ion Storage
Source: Nanomicro Lett. 2022 Jan 22;14:47. doi: 10.1007/s40820-022-00791-y (PMC8783942; doi:10.1007/s40820-022-00791-y)
Supplement: Supplementary file 1 — Supplementary file1 (PDF 2448 kb) [file 40820_2022_791_MOESM1_ESM.pdf]

Supporting Information for

Design of Flexible Films Based on Kinked Carbon Nanofibers for High Rate and Stable Potassium-Ion Storage

Qiaotian Xiong[1], Hongcheng He[1], and Ming Zhang[1, 2,] *

[1]Key Laboratory for Micro/Nano Optoelectronic Devices of Ministry of Education & Hunan Provincial Key Laboratory of Low-Dimensional Structural Physics & Devices,School of Physics and Electronics, College of Semiconductors (College of Integrated Circuits), Hunan University, Changsha 410082, P. R. China

[2]Semiconductor Technology and Application Innovation Institute of Changsha, Changsha 410012, P. R. China

*Corresponding author. E-mail: zhangming@hnu.edu.cn (Ming Zhang)

S1 Supplementary Equations

The fiber can be regarded as a cylinder with large aspect ratio. Based on the mechanics of materials, assuming that there is no extrusion between the longitudinal fibers and that each fiber is only subjected to unidirectional tension or pressure, the relationship between the normal stress and the linear strain on each fiber obeys Hooke's law:

$$\sigma = \frac{My}{I_z} \tag{S1}$$

The σ is the normal stress at any point of the cross section; M is the bending moment on the cross section; y is the ordinate of any point in the cross section; I_z is the moment of inertia of the cross section facing the neutral axis z.

At the microscopic level of the fiber's internal inserts, the relationship between shear modulus, elastic modulus and Poisson's ratio can be obtained by the following **Equation (S2)**:

$$G = \frac{E}{2(1+\mu)} \tag{S2}$$

If instead of a few tiny holes, the fiber has some other solid particles embedded in it, its shear modulus and bulk modulus are replaced by **Eqs. (S3)** and **(S4)** respectively to obtain [S1]:

$$\frac{G}{G_m} = \frac{1}{(1-c)^2}\left(\frac{3(1-v_m)}{4(1-2v_m)-(1-5v_m)(G/G_m)^{-3/5}}\right)^{1/3} \tag{S3}$$

$$\frac{K}{G} = \frac{4}{3} - \frac{2(1-5v_m)}{3(1-2v_m)}\left(\frac{G}{G_m}\right)^{-3/5} \tag{S4}$$

At the Macro-level of fabric size, the material in the domain of each contact point (C_p) inside the fiber is defined as the contact element E_C (C_p). The frictional work of the entangled network of nanofibers is shown as follows [S2]:

$$W_{cf}\left(E_C(c_k)\right) = R_N\left(gap\left(E_C(c_k)\right)\right)N + R_T\left([U]_T\left(E_C(c_k)\right)\right),$$

$$V^{(i)}\left(\xi^{(i)}\right) - V^{(j)}\left(\xi^{(j)}\right). \tag{S5}$$

Where R_N normal friction force is a function of gap

$$R_N(gap) = \begin{cases} 0 \quad (gap \geq 0); \\ \dfrac{K_N}{2g_r} gap^2 \quad (g_r \leq gap < 0); \\ K_N\left(gap + \dfrac{g_r}{2}\right) \quad (gap < g_r). \end{cases} \tag{S6}$$

And, the R_T tangential friction is a function of tangential relative displacement

$$R_T\left(E_C(c_k)\right) = \begin{cases} \dfrac{\mu \parallel R_N \parallel}{u_{T,rev}}[U]_T\left(E_C(c_k)\right) \quad \left([U]_T\left(E_C(c_k)\right) \leq u_{T,rev}\right); \\ \dfrac{\mu \parallel R_N \parallel}{\parallel [U]_T \parallel}[U]_T\left(E_C(c_k)\right) \quad \left([U]_T\left(E_C(c_k)\right) > u_{T,rev}\right). \end{cases} \tag{S7}$$

In the above formula, K_N is the compensation coefficient, g_r is the threshold of regularization coefficient, μ is the Coulomb friction coefficient, and $u_{T,rev}$ is the reversible tangential displacement.

For the discussion and simulation of fiber flexibility with kink trend, because it is similar to micro-spring structure, here we can intuitively show the flexibility of kink fiber with different effective turns and fiber radius. The stiffness coefficient k is usually also called the spring coefficient (**Eq. (S8)**). When k is larger, it means that the stiffness of the spring is about strong, and when k is about small, it means that the spring is about soft. It is easy to know from the formula that under the same meridian, the spring with larger effective turns and smaller wire diameter will be softer. It means that when the same horizontal external force is applied to the top after fixing one end of the spring, the smaller the fiber k, the greater the vertical displacement of the end point, which shows that it has better flexibility.

$$\mathrm{k} = \frac{G \times d^4}{8N_c \times {D_m}^3} \tag{S8}$$

S2 Supplementary Figures and Tables

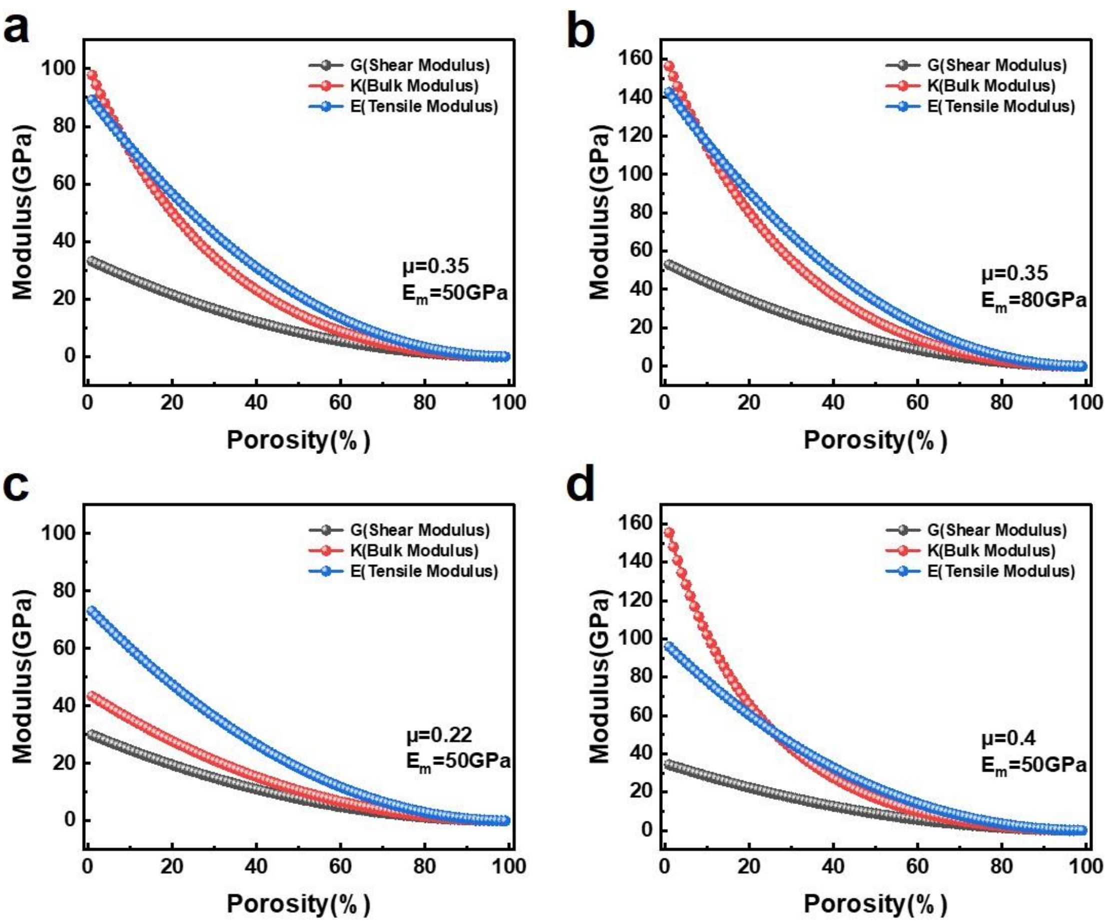

Fig. S1 variation relationship of bulk modulus, shear modulus and elastic modulus with porosity in different elastic modulus (**a**) and (**b**) or different Poisson's ratio (**a**) (**c**) and (**d**)

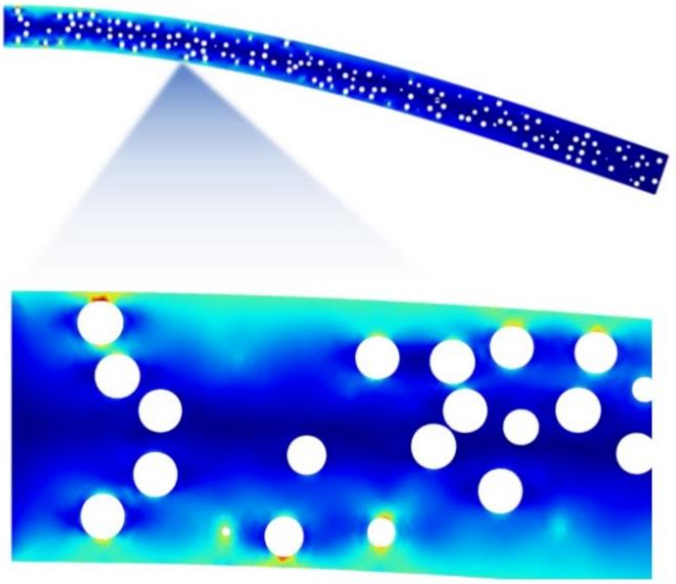

Fig. S2 The schematic diagram shows that if there are too many mesopores and the mesopores are located on or near the surface, the Stress Singularity will occur when bending deformation

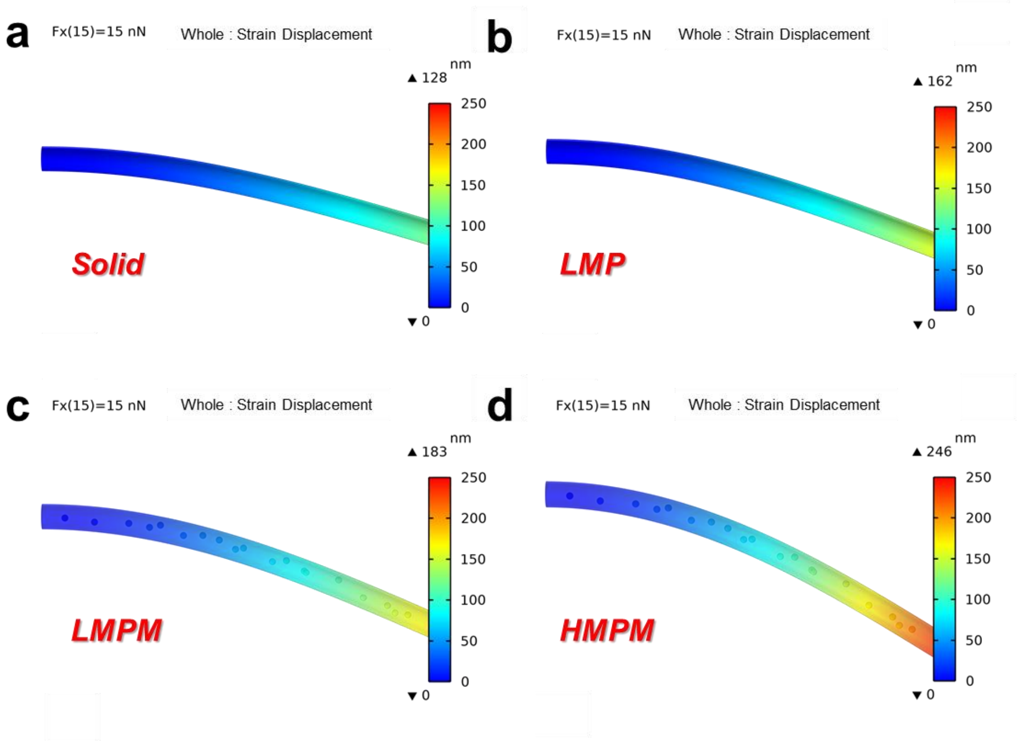

Fig. S3 Schematic diagram of overall displacement of solid fiber (non-porous), low microporous porosity fiber (LMP), high microporous porosity fiber (HMP) and high microporous porosity inner porous fiber (HMPM) when the external load force is 15 nN

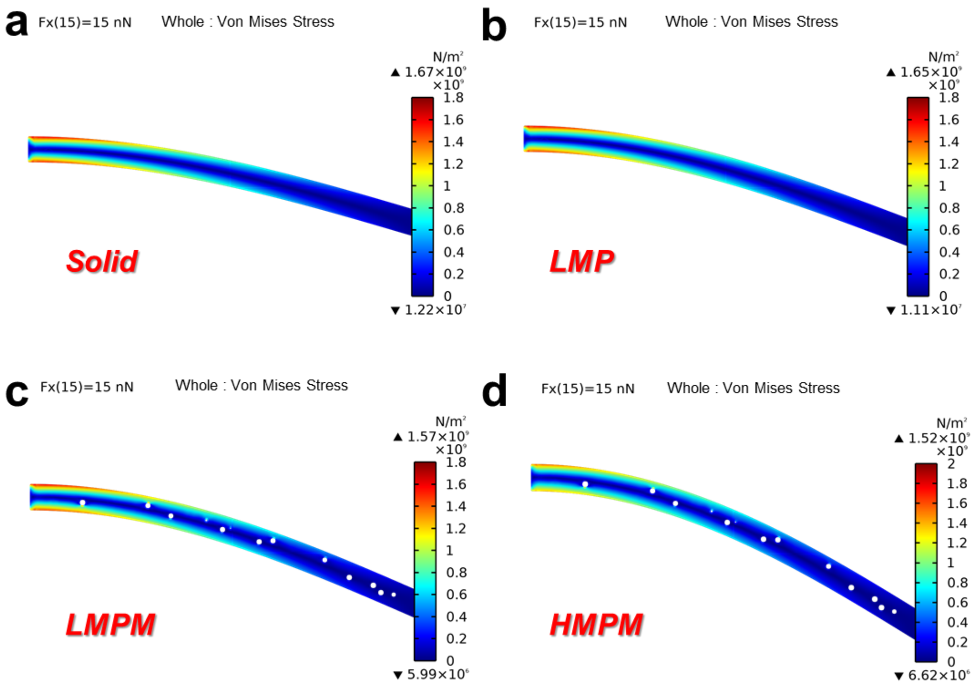

Fig. S4 Schematic diagram of sectional stress distribution of solid fiber (non-porous), low microporous porosity fiber (LMP), high microporous porosity fiber (HMP) and high microporous porosity inner porous fiber (HMPM) when the external load force is 15 nN

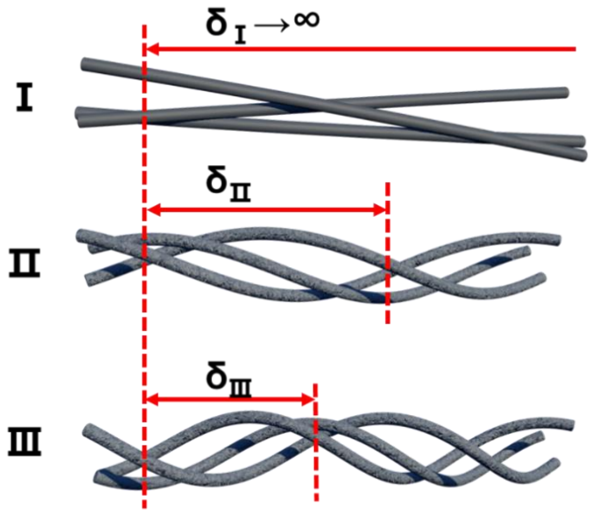

Fig. S5 Expression of the degree of kink: the smaller the distance between two kinks (δ), the greater the degree of kink. I is kink free in the ideal state, so δ_I tends to infinity; δ_{III} is smaller than δ_{II}, so III has a greater degree of kink

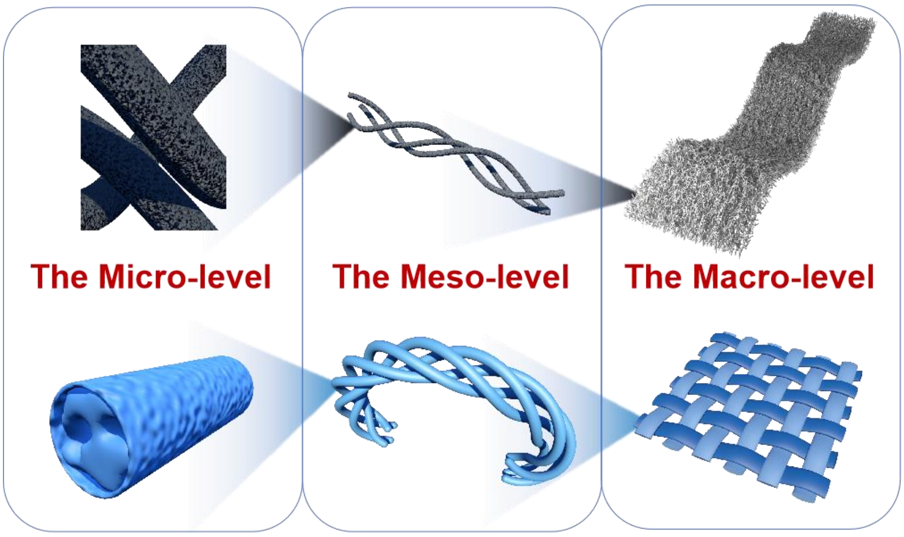

Fig. S6 The illustrations are used to explain the origin and essence of the flexibility of doped micro kink carbon nanofiber felt from the micro-, meso- and macro-levels

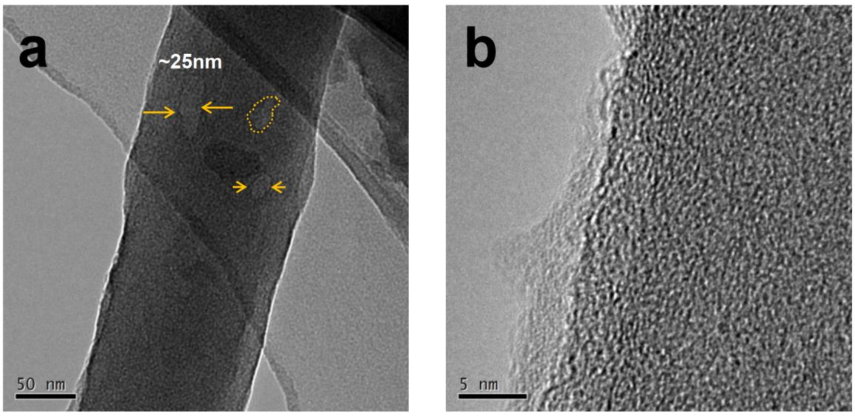

Fig. S7 (**a**) TEM image, (**b**) HRTEM image corresponding to S/N-KCNFs

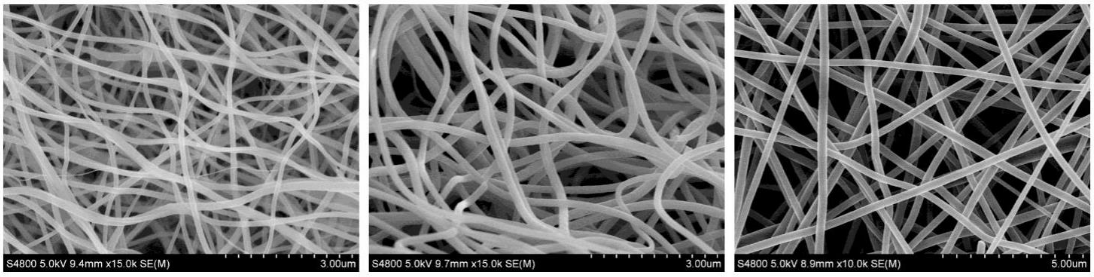

N-KCNFs S-KCNFs CNFs

Fig. S8 The SEM images of the N-doped and S-doped micro kinked carbon nanofibers, respectively; and undoped carbon nanofibers

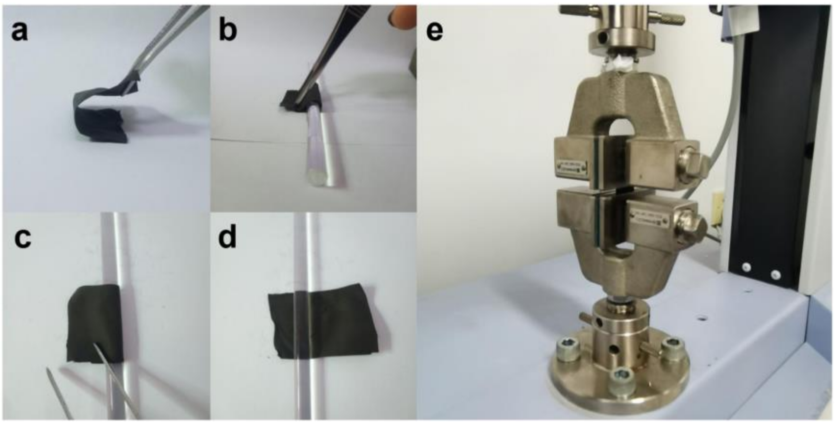

Fig. S9 (**a-d**) Digital photos of S/N-KCNFs bending and automatic restoration; (**e**) Felts were measured by precision universal testing machine (Shimadzu AGS-X)

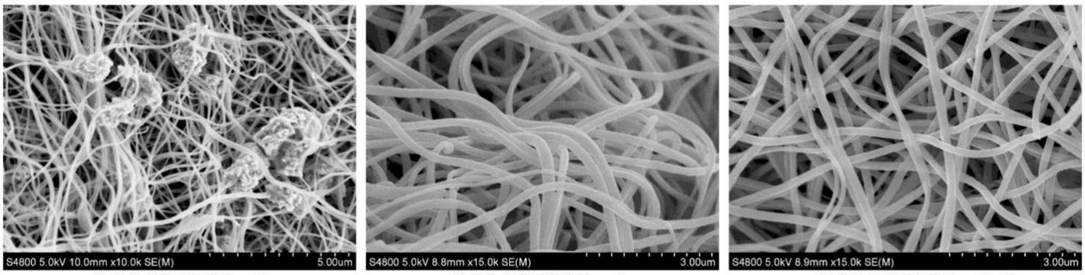

S/N-KCNFs-2 S/N-KCNFs-1 S/N-KCNFs-0.5

Fig. S10 SEM images of PAN: MA mass ratio in S/N-KCNFs were changed to 1:2 and 2:1

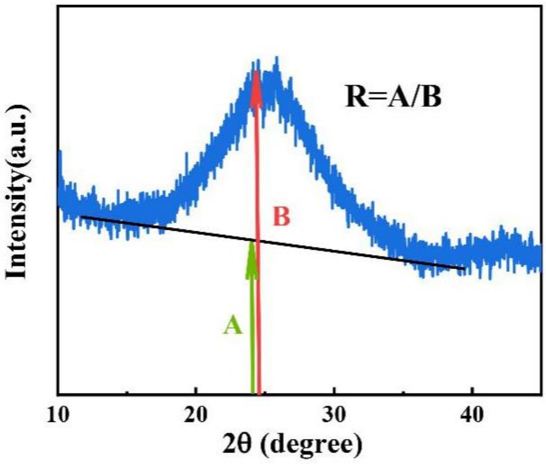

Fig. S11 The R value is A/B in the XRD curve to indicate the degree of carbon defects

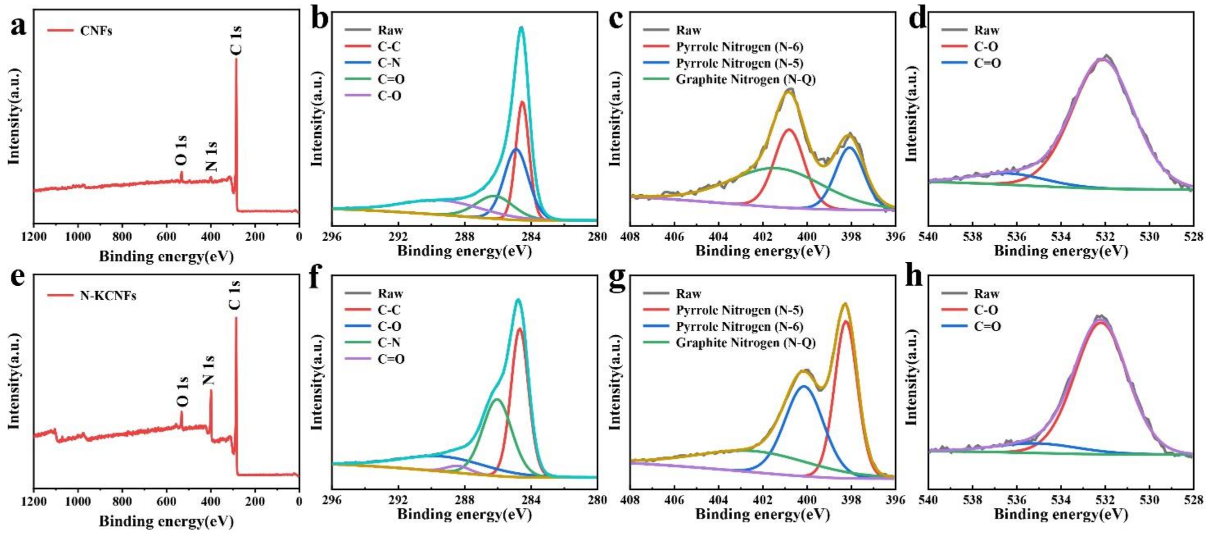

Fig. S12 In XPS, the full spectrum (**a**), C 1s (**b**), O 1s (**c**) and N 1s (**d**) of CNFs; The full spectrum (**e**), C 1s (**f**), O 1s (**g**) and N 1s (**h**) of N-KCNFs were obtained by adding melamine

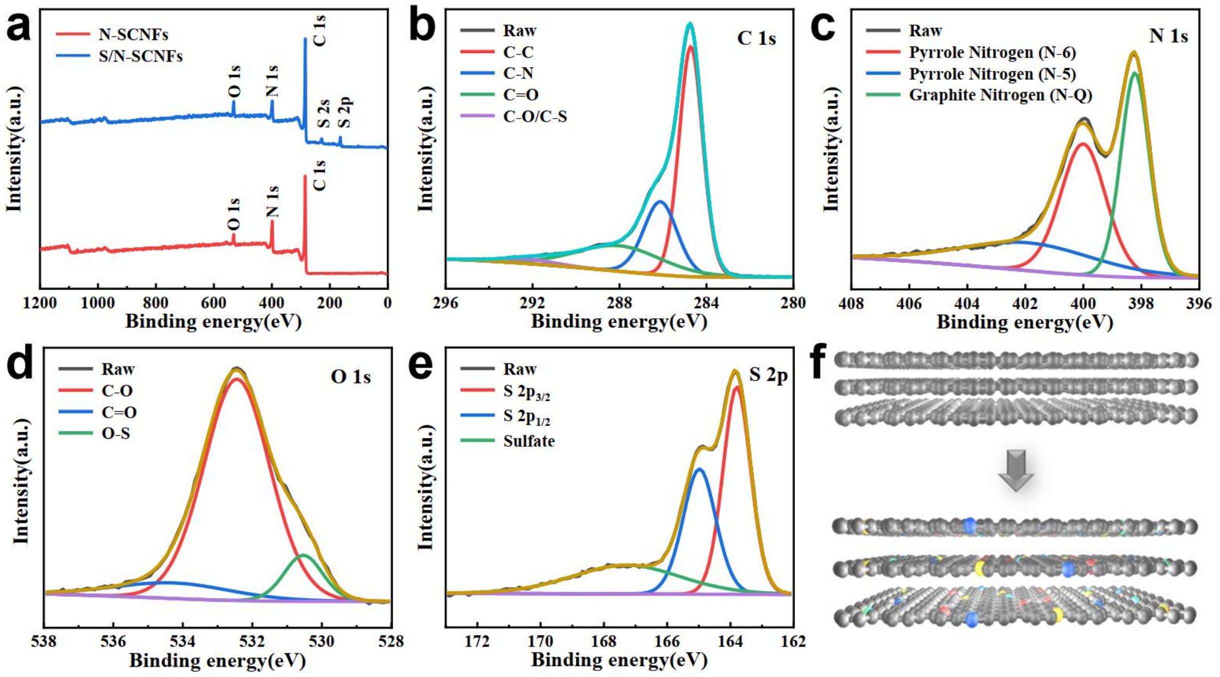

Fig. S13 (**a**) XPS measurement spectra of S/N-KCNFs, N-KCNFs; High-resolution (**b**) C 1s, (**c**) N 1s, (**d**) O 1s and (**e**) S 2P spectra of S/N-KCNFs; (**f**) Schematic illustration of heteroatom doping in carbon layer

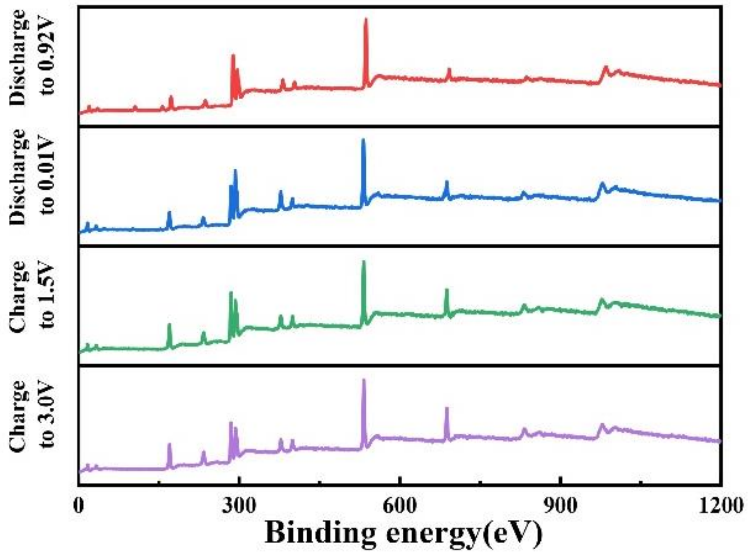

Fig. S14 Ex-situ XPS of different periods in charge discharge process: discharge to 0.92 V, discharge to 0.01 V, charge to 1.5 V, charge to 3.0 V

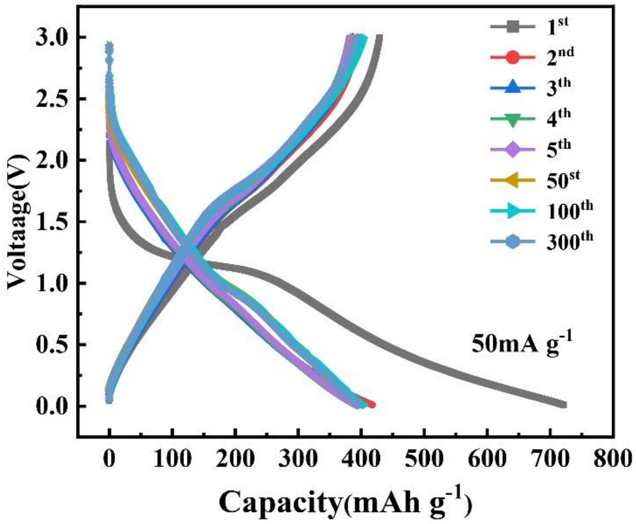

Fig. S15 The voltage specific capacity curves of S/N-KCNFs as anode in the first 5 cycles, the 50th cycle, the 100th cycle and the 300th cycle under the current density of 50 mA g^{-1} were obtained

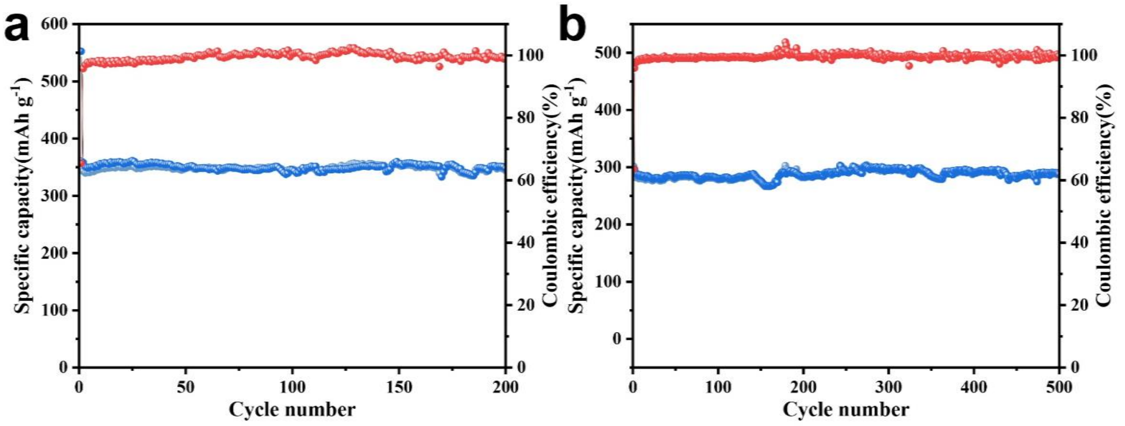

Fig. S16 The results are the cyclic stability test of S/N-KCNFs at 300 mA g^{-1}(**a**) and 800 mA g^{-1} (**b**)

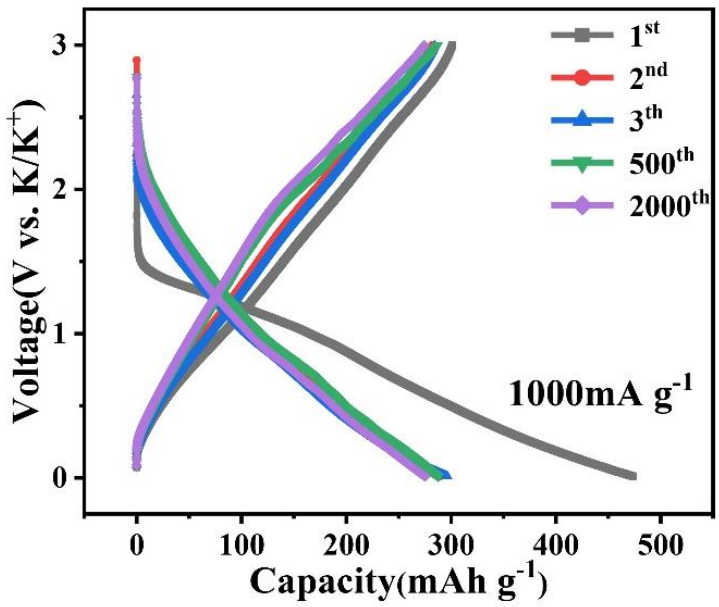

Fig. S17 The voltage specific capacity curves of S/N-KCNFs as anode in the first 3 cycles, the 500th cycle and the 2000th cycle under the current density of 1000 mA g^{-1} were obtained

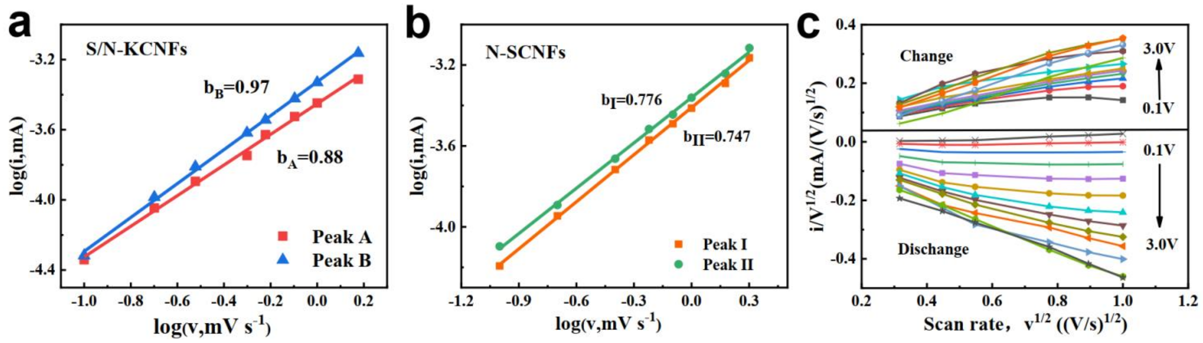

Fig. S18 The curves of logarithm (I) and logarithm (V) of S/N-KCNFs (**a**) and N-KCNFs (**b**) at the cathode/anode peak pair (peak current: I, scanning rate: V); (**c**) The curves of $v^{1/2}\ vs. I/v^{1/2}$ used to calculate constants K$_1$ and K$_2$ at different potentials

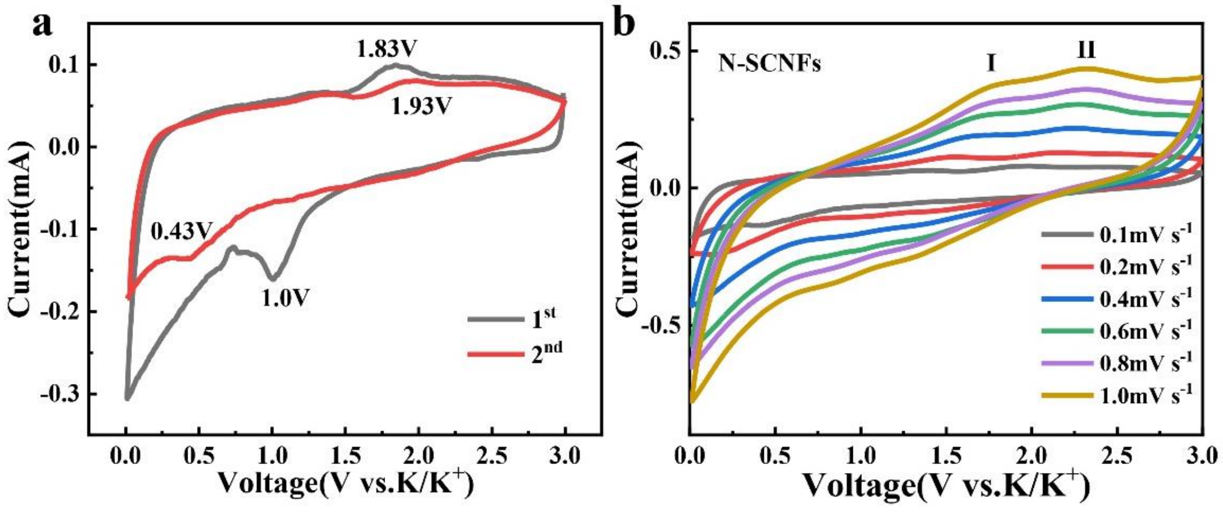

Fig. S19 (**a**) The CV Curve of the first two cycles of N-KCNFs; (**b**) The CV Curve of N-KCNFs in the scanning rate range of 0.1-1 mV s^{-1}

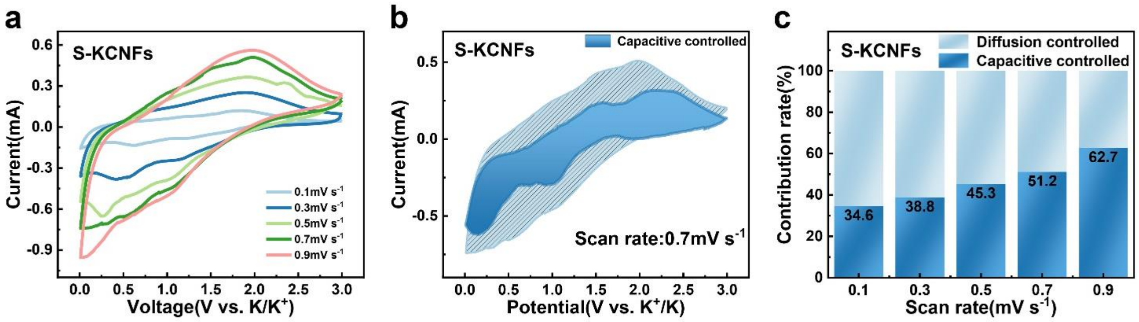

Fig. S20 The respective capacitance contributions of S-KCNFs electrodes

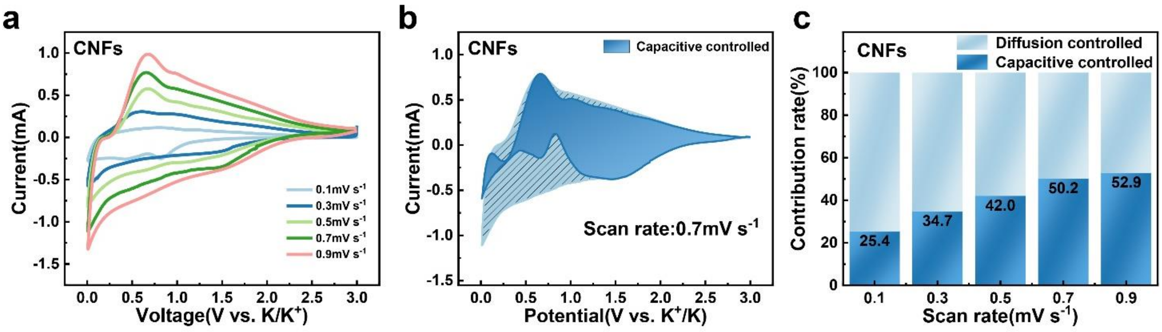

Fig. S21 The respective capacitance contributions of CNFs electrodes

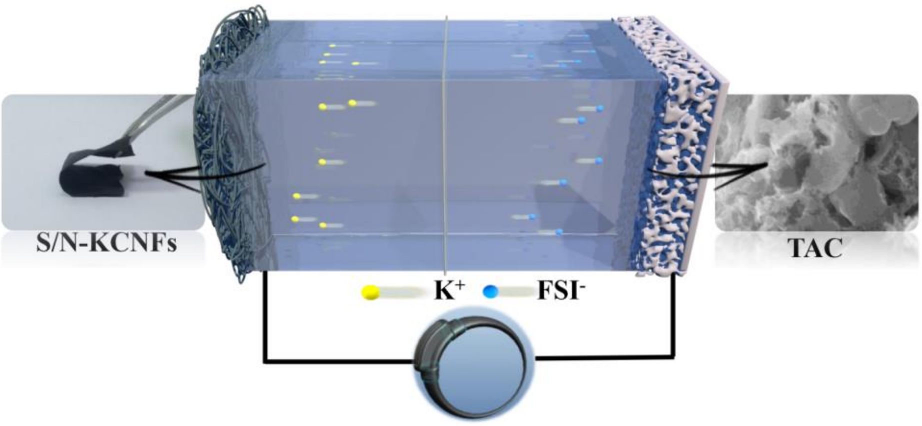

Fig. S22 Schematic illustration of hybrid capacitor S/N-KCNFs//TAC supplying power to wearable flexible devices

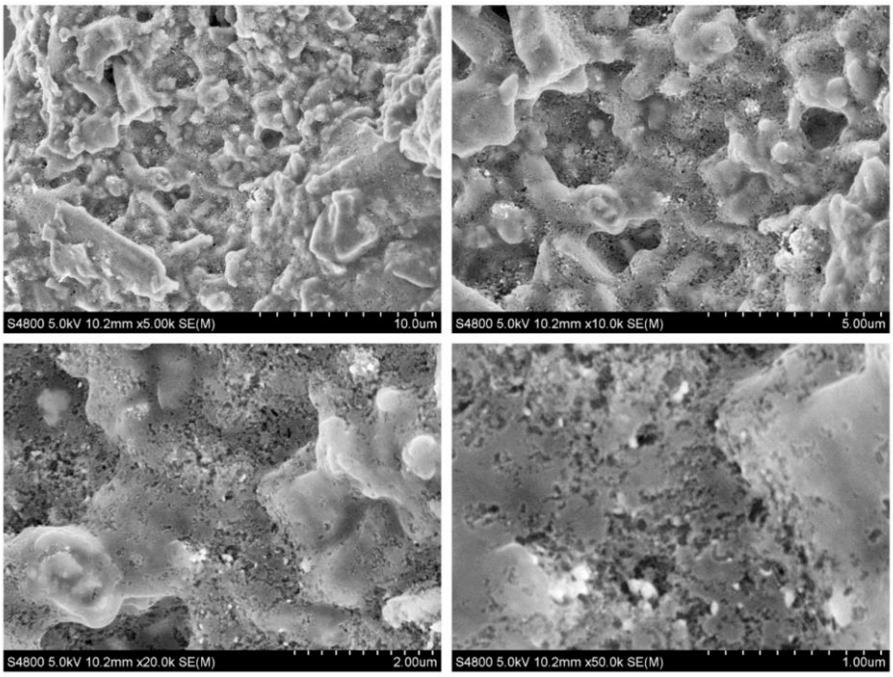

Fig. S23 Different magnification of SEM illustration of activated carbon treated by dilute nitric acid and high temperature

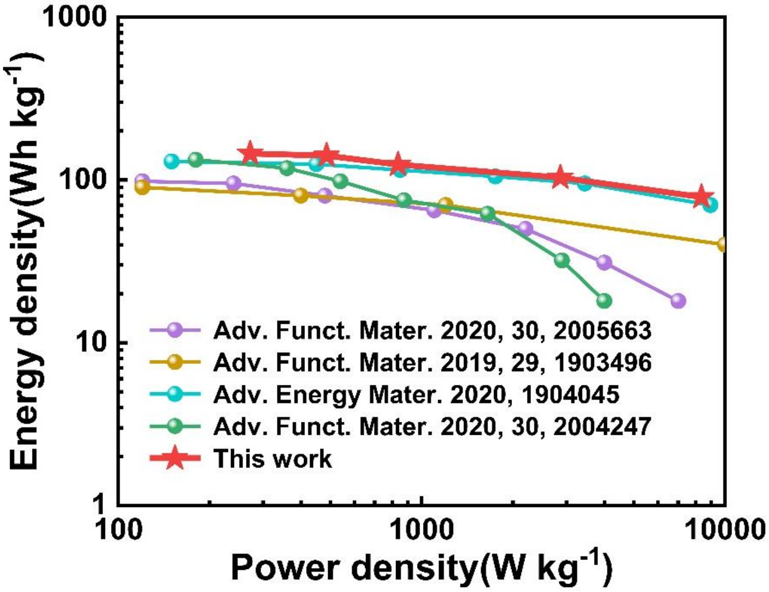

Fig. S24 Ragone plots of the S/N-KCNFs//TAC and other similar works in PIHCs [S3-S6]

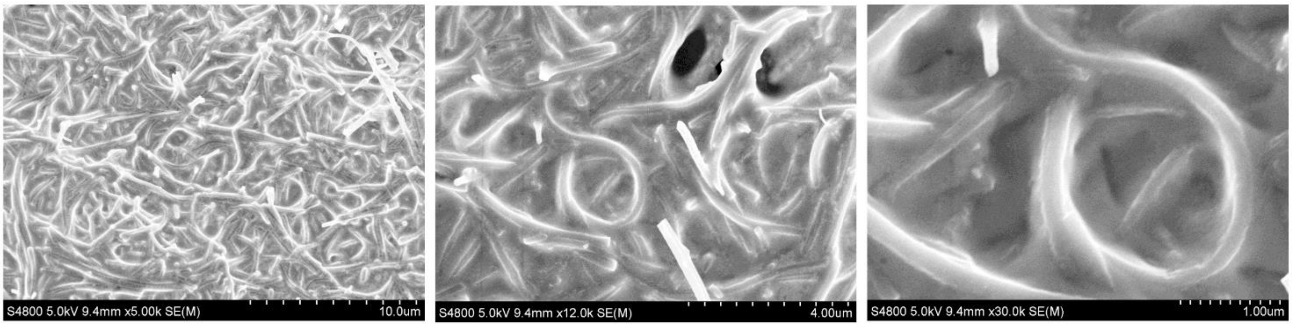

Fig. S25 Different magnification of SEM illustrations of the reacted S/N-KCNFs surface obtained after the recycling battery was disassembled

Table S1 The N content of different PAN:MA materials was obtained by the element analysis in XPS

Element content ratio	Mass ratio of PAN: MA			
	1:0	2:1	1:1	1:2
N:C	3.8%	13.4%	16.7%	22.3%

Table S2 The content of N in the materials at different temperatures was determined by XPS

Element content ratio	Different temperature (℃) in PAN: MA =1: 1		
	600	700	800
N:C	18.3%	16.7%	13.4%

Table S3 Atomic percentage of C, N, O and S in N/S-KCNFs, N-KCNFs and CNFs was determined by XPS

Materials	Element（at.%）by XPS			
	C 1s	O 1s	N 1s	S 2p
KCNF	94.77	2.78	2.45	/
N-KCNF	80.07	2.94	16.99	/
N/S-KCNF	80.53	2.77	13.31	3.39

Table S4 Percentage of three types of nitrogen doped carbon-based materials in N/S-KCNFs, N-KCNFs and CNFs: pyrrole nitrogen (N-5), pyrrole nitrogen (N-6) and graphite nitrogen (N-Q)

Materials	pyrrole nitrogen (N-5)	pyrrole nitrogen (N-6)	graphite nitrogen (N-Q)
KCNF	22.86	30.20	46.95
N-KCNF	37.67	36.72	25.61
N/S-KCNF	34.05	41.41	24.53

Table S5 The percentage of each element in different periods of charge discharge process was obtained by ex- situ XPS

Materials	Element（at%）by XPS				
	C 1s K 2p	O 1s	N 1s	S 2p	F 1s
Discharge to 0.92V	39.26	38.56	6.84	10.09	5.25
Discharge to 0.01V	39.18	33.36	8.18	11.93	7.35
Charge to 1.5V	51.22	22.33	6.35	11.00	9.09
Charge to 3.0V	36.53	29.45	8.24	14.03	11.74

Table S6 Specific surface area and pore volume volumes in N/ S-KCNFS, N-KCNFS, and CNFs in BET tests, as well as the respective porosity obtained using a density of 1.75

Materials	N/S-KCNFs	N-KCNFs	CNFs
BET Surface Area (m/g)	340.0227	94.1202	23.4245
Pore Volume (cm^3/g)	0.196092	0.070309	0.029903
Porosity (%)	11.205	4.0176	1.7087

Supplementary References

[S1] R.W. Zimmerman, Elastic moduli of a solid containing spherical inclusions. Mech. Mater. **12**(1), 17-24 (1991). https://doi.org/ 10.1016/0167-6636(91)90049-6

[S2] D. Durville, Numerical simulation of entangled materials mechanical properties. J. Mater. Sci. **40**(22), 5941-5948 (2005). https://doi.org/10.1007/s10853-005-5061-2

[S3] J. Ruan, F. Mo, Z. Chen, M. Liu, S. Zheng et al., Rational construction of nitrogen-doped hierarchical dual-carbon for advanced potassium-ion hybrid capacitors. Adv. Energy Mater. **10**(15), 1904045 (2020). https://doi.org/10.1002/aenm.201904045

[S4] D. Qiu, J. Guan, M. Li, C. Kang, J. Wei et al., Kinetics enhanced nitrogen-doped hierarchical porous hollow carbon spheres boosting advanced potassium-ion hybrid capacitors. Adv. Funct. Mater. **29**(32), 1903496 (2019). https://doi.org/10.1002/adfm.201903496

[S5] Y.Z. Fang, R. Hu, K. Zhu, K. Ye, J. Yan et al., Aggregation-resistant 3D $Ti_3C_2T_x$ MXene with enhanced kinetics for potassium ion hybrid capacitors. Adv. Funct. Mater. **30**(50), 2005663 (2020). https://doi.org/10.1002/adfm.202005663

[S6] M. Chen, L. Wang, X. Sheng, T. Wang, J. Zhou et al., An ultrastable nonaqueous potassium-ion hybrid capacitor. Adv. Funct. Mater. **30**(40), 2004247 (2020). https://doi.org/10.1002/adfm.202004247
